# Serosurveillance for Measles and Rubella

**DOI:** 10.3390/vaccines12070816

**Published:** 2024-07-22

**Authors:** Allison M. Brady, Elina El-Badry, Eriko Padron-Regalado, Nicole A. Escudero González, Daniel L. Joo, Paul A. Rota, Stephen N. Crooke

**Affiliations:** Division of Viral Diseases, National Center for Immunization and Respiratory Diseases, Centers for Disease Control and Prevention, Atlanta, GA 30333, USAjtp0@cdc.gov (D.L.J.); par1@cdc.gov (P.A.R.)

**Keywords:** immune surveillance, serosurvey, seroprevalence, measles, rubella, multiplex assay, IgG antibody

## Abstract

Measles and rubella remain global health threats, despite the availability of safe and effective vaccines. Estimates of population immunity are crucial for achieving elimination goals and assessing the impact of vaccination programs, yet conducting well-designed serosurveys can be challenging, especially in resource-limited settings. In this review, we provide a comprehensive assessment of 130 measles and rubella studies published from January 2014 to January 2024. Methodologies and design aspects of serosurveys varied greatly, including sample size, assay type, and population demographics. Most studies utilized enzyme immunoassays for IgG detection. Sample sizes showed diverse sampling methods but favored convenience sampling despite its limitations. Studies spanned 59 countries, predominantly including adults, and revealed disparities in seroprevalence across demographics, regions, and notably among migrants and women. Age-related declines in antibodies were observed, particularly among infants, and correlations between vaccination status and seropositivity varied. We conclude with an outlook on measles and rubella serosurveillance, emphasizing the need for proper survey design and the advantages of standardized, multiplex serology assays.

## 1. Introduction

### 1.1. Measles and Rubella: Epidemiology, Disease Burden, and Elimination Efforts

Measles and rubella continue to pose significant public health challenges despite the availability of effective vaccines and global efforts towards their elimination. Measles, caused by the measles virus, is a highly contagious disease characterized by fever, cough, coryza, conjunctivitis, and a distinctive rash, which can lead to severe health complications, particularly in unvaccinated people [1]. Rubella, often milder in children and adults, can have devastating consequences if contracted during early pregnancy, leading to congenital rubella syndrome (CRS) in the developing fetus. This syndrome can include a range of severe birth defects or fetal deaths [2]. Two doses of the measles-containing vaccine (MCV) and a single dose of the rubella-containing vaccine (RCV) can provide lifelong protection [3,4,5]. Moreover, high levels of measles and rubella antibodies within a population contribute to herd immunity, which helps protect individuals who are unable to be vaccinated (e.g., infants, pregnant women, and immunocompromised individuals) by reducing the overall transmission of the viruses. Despite the availability of effective vaccines for measles, the incidence of measles doubled between 2017 and 2018, a trend that continued into 2019, leading several countries to lose their measles elimination status [6]. In 2022, measles incidence trended upwards, with an estimated 136,200 deaths and 9.2 million cases, marking a 43% increase in mortality and an 18% increase in cases compared with 2021 [7]. Rubella cases dropped by 48% between 2012 and 2019 and continued to decline into 2020. However, CRS cases increased between 2012 and 2022, which can be attributed to the initiation of CRS surveillance in several large countries [8]. It is estimated that 32,000–100,000 cases of CRS occur annually, depending on the estimate model used, underscoring the persistent public health challenge posed by rubella infection [5,9,10,11].

To facilitate concerted efforts towards elimination, the Measles and Rubella Initiative devised the Global Measles and Rubella Strategic Plan 2012–2020, aligning with the WHO’s Global Vaccine Action Plan 2011–2020 [12]. As part of this strategic plan, measles and rubella elimination was targeted in at least five WHO regions by the end of 2020 through the implementation of five core strategies [8,13]. Ultimately, complete elimination goals were not met by 2020 [14], though some progress was made. Between 2000 and 2019, global measles-containing vaccine first dose (MCV1) coverage increased from 72% to 86% [7]. Global routine measles-containing vaccine second dose (MCV2) coverage has increased steadily, from 42% in 2010 to 69% in 2018. By the end of 2019, 178 WHO member states had taken the critical step of introducing MCV2, underscoring the collective commitment towards advancing immunization initiatives and progress towards measles elimination [15]. Between 2012 and 2022, the number of countries providing rubella-containing vaccine (RCV) increased from 132 to 175, and verification of rubella elimination was documented in 98 (51%) of 194 WHO member states [8]. Unfortunately, the disruption of routine vaccination programs and supplemental immunization activities (SIAs) during the COVID-19 pandemic led to a decline in MCV1 coverage to 81% in 2021, the lowest since 2008 [7,16,17]. MCV1 coverage has begun to recover but remains alarmingly low (83% in 2022) [7]. Despite the fact that immunization averted approximately 23 million deaths between 2010 and 2018, the continued transmission of measles remains a potent reminder of the importance of sustaining robust immunization programs and surveillance systems to prevent outbreaks.

To continue efforts towards elimination, the Measles and Rubella Strategic Framework (MRSF) 2021–2030 was developed to provide a comprehensive guide on supporting global, regional, and national efforts. MRSF emphasizes strategic priorities such as integrating efforts into primary health care and universal health coverage, enhancing surveillance systems, ensuring vaccine supply, and promoting research and innovation. This framework aligns with the Immunization Agenda 2030 (IA2030) [18]. IA2030 outlines a global strategy to enhance health through immunization, in which measles and rubella elimination play a critical role, positioning measles as a “tracer” for the effectiveness of immunization programs. Measles outbreaks may indicate gaps in routine childhood immunization coverage [19]. Regardless, achieving high uniform vaccination coverage for measles and rubella remains a challenge, particularly in resource-constrained regions, and continues to impede progress towards elimination. Even for countries that have good overall coverage rates, immunization coverage in-country can vary greatly due to regional healthcare disparities, inadequate vaccine supply chain, political or social instability, or inadequate monitoring and surveillance [20]. Collectively, these challenges underscore the critical importance of enhancing surveillance systems and leveraging serosurveillance tools to monitor and address gaps in immunity. This approach can help guide the design of effective vaccination strategies aimed at preventing outbreaks and advancing toward the goal of measles and rubella elimination.

### 1.2. Antibody Response and Immune Memory to Measles and Rubella

The WHO currently recognizes eight clades of measles (A-H); however, there is only one serotype [21]. For rubella, there are thirteen different genotypes divided into two clades (1a, 1B, 1C, 1D, 1E, 1F, 1G, 1h, 1i, 1j, 2A, 2B, and 2C); however, like measles, there is only one serotype [22]. Upon initial exposure to a virus, either through disease or vaccination, the immune system initiates a primary immune response, leading to the production of various classes of antibodies. Immunoglobulin M (IgM) antibodies are prevalent early during infection. During measles infection, IgM levels peak a few days after the onset of the rash and decrease in the subsequent weeks. Similarly, during a rubella infection, IgM is typically detectable within two to five days after rash onset and remains such for one to three months post-infection. IgM levels decline as B cells undergo class switching to produce immunoglobulin G (IgG) antibodies. Measles IgG levels peak one to two weeks after the rash onset, although low levels are detectable within a few days after the rash appears [23,24]. Anti-rubella IgG becomes detectable shortly after IgM, peaking around one to two weeks after the rash appears [25]. IgG antibodies serve as unique biomarkers of prior exposure (either by vaccination or infection) to measles and rubella, and they typically persist for life [23]. The differentiation of B cells into memory B cells is crucial for producing high-affinity IgG antibodies and establishing a sustained antibody-mediated immune response post-exposure. Additionally, memory B cells can terminally differentiate into long-lived, antibody-producing plasma cells, usually conferring protection during subsequent re-exposure to a pathogen [26]. Measles and rubella vaccines are live, attenuated viruses that cause a similar infection to wild-type viruses, although usually without symptoms. In general, the antibody response is less robust to immunization than to disease and results in lower antibody titers. However, antibody tests cannot distinguish between disease-induced and vaccination-induced antibodies.

In addition to antibody production following infection with a wild-type or vaccine virus, pathogen-specific antibodies can be transferred from one person to another in two ways. The first is prophylactic administration of pooled human immune globulin with sufficient pathogen-specific antibodies by intramuscular, intravenous, or subcutaneous injection [27]. This is a rare procedure, and the antibody duration is only a few months, so this seroprevalence is irrelevant on a population scale. The second method of antibody transfer occurs during pregnancy, when a mother transfers her own antibodies through the placenta to her baby. The titer achieved in the infant is dependent on the mother’s titer and is typically roughly two-fold higher. On average, infants whose mothers have natural infection-induced immunity have higher titers than infants whose mothers have vaccine-induced immunity [28,29]. Infants of mothers who have no measles antibodies do not receive this transplacental protection. The duration of antibody protection among infants is directly related to the titer at birth and is decreasing across infant populations because of increasing proportions of mothers who have vaccine-induced or no measles immunity [29,30,31]. Because of the low levels of maternally derived measles antibodies and the severity of measles in infants, seroprevalence studies in infants represent a special case of serosurveys and need to be analyzed separately, typically by months of age.

In general, the measles seroprevalence in a population is highly age-dependent. Almost all infants are born with measles antibodies (provided their mother was previously vaccinated or infected), so the seroprevalence in newborns is close to 100%. However, titers in infants decrease rapidly, and, in many settings, infant titers are near zero by 4 to 6 months of age [32,33,34,35]. This is not a major risk in countries with elimination status because the likelihood of exposure to measles is very low. However, in measles-endemic countries, early infant susceptibility has led to increases in measles incidence among infants [36]. After maternal antibodies are depleted, measles seroprevalence in infants begins to increase as they are either vaccinated or infected with measles. Unfortunately, it is not possible to distinguish between maternally transferred, disease-induced, or vaccine-induced antibodies using serological testing.

The detection of IgG in serum does not necessarily indicate the presence of protective, neutralizing antibodies [37]. Functional, or neutralizing, antibodies can bind specific epitopes required for viral entry into host cells, thus preventing subsequent infections. Therefore, neutralizing antibodies are reliable indicators of protective immunity [38]. For measles, a neutralizing antibody titer of 120 milli-International Units (mIU)/mL is well established to protect against disease, although there has been some debate surrounding this in recent years [39]. The accepted correlate of protection for rubella is 10 IU/mL [40]. IgG antibodies that lack neutralizing activity (e.g., binding antibodies) are also produced during an immune response. While binding antibodies are pathogen-specific and may have other effector functions, they lack the ability to neutralize an infectious virus. Serological surveys typically assess the overall prevalence of IgG antibodies against specific pathogens in the population, irrespective of antibody activity [26].

In addition to humoral immunity, exposure to a pathogen triggers a cellular immune response. Broadly, CD4+ T cells provide essential help for several immune functions, such as affinity maturation of B cells and antibody isotype switching, while CD8+ T cells commonly exert antiviral effects by producing cytokines that suppress virus replication and eliminate infected cells. In the response to measles virus infection, T cells are considered essential for viral clearance [41]. Individuals with low or undetectable measles antibody levels have been shown to be somewhat protected against clinical measles, suggesting a significant role for cellular immunity [42]. However, the correlation between measles-specific antibody levels and the T cell immune response is poor [43]. Attempts to establish a cellular correlate of protection for rubella have failed to date [40], and no association between markers of humoral and cellular immunity has been identified [44]. Although seroprotective antibody titers for measles and rubella have been established, these correlates of protection should not be used to assess or infer the protection conferred by the cellular immune response. Additional studies are needed to establish correlates of protection specific to the cellular immune response.

### 1.3. Serosurveillance

Serosurveys are critical for understanding population-level immunity and disease burden estimates for infectious diseases. Typically, serosurveys do this by monitoring the presence of pathogen-specific IgG antibodies. For vaccine-preventable diseases (VPDs), well-designed serological surveillance studies can aid in assessing the effectiveness of vaccination programs and help identify gaps in immunity. For non-VPDs or for VPDs in countries where vaccination is not established, serosurveillance data can estimate disease burden or changes in disease exposure [45]. Unfortunately, serosurveys for estimating the disease burden of VPD can be complicated in areas with vaccination, as disease- and vaccine-induced antibodies are measured jointly and cannot be distinguished. These data can collectively guide public health decisions on vaccination policies and strategies to achieve and maintain elimination. For measles and rubella, serological surveys have been instrumental in global efforts towards elimination by evaluating immunity to identify susceptible populations or age groups, estimating disease burden in areas without vaccination, and assessing the impact of public health policies and campaigns [46]. Rubella serosurveys have also provided insights into true estimates of CRS disease burden [47].

Serosurveys often utilize enzyme immunoassays (EIAs) to assess the presence of IgG antibodies. Some EIAs and other IgG binding assays measure the total IgG antibody concentration, while others only provide a qualitative result, indicating the presence or absence of pathogen-specific IgG. EIAs can vary greatly in their sensitivity and specificity, depending on the antigen used (e.g., purified protein or whole virus lysate) as well as the assay format (e.g., direct, indirect, sandwich, or competitive EIA). These differences in assay performance can impact the ability to detect antibodies, particularly at low concentrations. One drawback of EIAs is the inability to distinguish neutralizing antibodies since these assays measure IgG binding to specific epitopes, regardless of their neutralization capacity [48]. For measles, the most abundantly produced antibodies target measles nucleoprotein, making it a common target in commercially available assays; however, nucleoprotein-specific antibodies are generally non-neutralizing; thus, total IgG titers do not correlate well with neutralization titers [48]. For rubella, the E1 glycoprotein is commonly used as the target for commercial test kits and is a target of neutralizing antibodies. EIA tests that provide a qualitative titer for rubella are designed to correspond with a protective level of antibodies; however, many studies have shown a lack of standardization [49,50,51,52,53]. Over time, antibody levels induced by measles and rubella vaccination may decrease [54,55]. As antibody levels wane, they may fall below the detection threshold for EIA assays that have poor sensitivity.

While EIAs are considered the standard methods for serosurveys, several studies have shown that EIAs and other immunostaining assays are less sensitive than plaque reduction neutralization tests (PRNTs), especially in the context of low antibody levels [56,57,58]. For measles, the plaque reduction neutralization test (PRNT) is considered the gold standard for assessing neutralizing immunity [59], while the immunocolorimetric neutralization assay is used for rubella [60]. A study by Tischer et al. demonstrated disparate positivity rates for measles between EIA, indirect immunofluorescence tests (IFT), and PRNT when serum samples were tested by each method [58]. The samples were collected from patients with known measles vaccination status but showed equivocal or negative results when initially tested with EIA several years after vaccination. Subsequent re-testing of these samples with IFT and PRNT showed positive rates of 81% for IFT and 94% for PRNT [58]. This discrepancy suggests that some EIAs may lack sufficient sensitivity to detect vaccine-induced antibodies several years post-immunization, posing potential challenges for serosurveys. However, applying neutralization assays in large-scale serosurveys is impractical due to technical difficulty, high cost, and labor-intensive processes.

Bead-based immunoassays have been around for more than two decades [61] and utilize antigen-bound beads to detect pathogen-specific IgG. Moreover, bead-based assays are often multiplexed, allowing for the simultaneous detection of antibodies to multiple pathogens in a single test. Bead-based immunoassays have been utilized for serological surveys for a variety of pathogens, including measles and rubella, and generally have robust performance characteristics, are cost-effective, and are resource-efficient [62]. A study by the National Institute of Public Health and the Environment in the Netherlands indicated a multiplexed bead-based assay (MBA) had higher sensitivity for measles and rubella IgG than individual ELISA [63]. MBA data exhibited strong correlations with ELISA data for both measles and rubella, with no cross-interference among antigens observed. Notably, MBA also demonstrated a higher correlation with PRNT than EIA [56,64]. In conclusion, MBAs present a compelling alternative to conventional EIA for detecting multiple target antibodies simultaneously in large-scale immunosurveillance studies.

## 2. Literature Review of Measles and Rubella Serosurveys (January 2014–January 2024)

### 2.1. Measles and Rubella Serosurvey Study Selection and Characteristics

**Figure 1:**
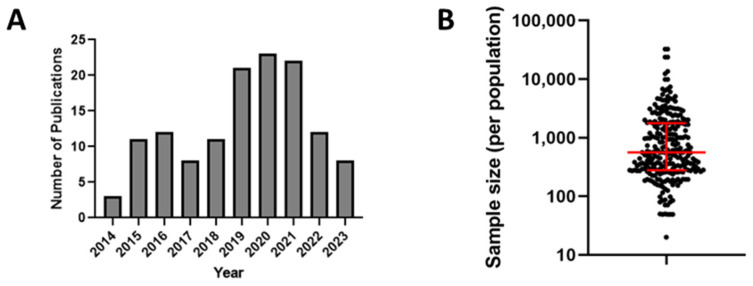
(**A**) Distribution of the number of publications by year included in the review. (**B**) Plot of serosurvey sample sizes for all included studies for measles and rubella. Red lines represent the median and interquartile range.

To understand the scope of measles and rubella seroprevalence surveys over the last decade, we conducted an online search using OVID (Medline, Embase, and Global Health), Cochrane Library, CINAHL, and Scopus. The search was constrained to the last 10 years (January 2014 through January 2024) and included the following keywords: ‘rubella’ and ‘measles’ combined with ‘serosurvey’, ‘seroprevalence’, ‘immunity’, and ‘population immunity’. All search sources were last consulted on 9 January 2024. Any duplicate articles returned by the search were removed, leaving 492 results. We further excluded references that were not in English or where a full-length, peer-reviewed primary research paper or relevant seroprevalence information were not found. Additionally, any studies that focused specifically on individuals with HIV or other chronic health conditions were also excluded. This resulted in a total of 130 published studies, of which 30 described serosurveys for measles, 25 for rubella, and 75 for both. We focused our analysis on distinct populations (e.g., age, sex, occupation, or pregnancy status) and reported seroprevalence for each population individually within the paper. We also grouped studies by year of publication, country, WHO region, and vaccination status. When appropriate, medians were used to calculate the central tendency of a research outcome. Records not showing these data items were considered missing or unavailable. We did not discriminate between studies using residual samples and studies using a priori study design for estimating some outcomes in our review (e.g., overall seroprevalence). Therefore, we acknowledge that this might influence these estimations, although the extent of such influence is unknown. Supplementary Tables S1 and S2 list each population described for each published study. A 2016 review of measles and rubella seroprevalence studies published between 1998 and June 2014 found a total of 68 studies for measles and 58 for rubella [65]. In comparison, there seems to be a notable increase in the publication rate of such studies (Figure 1A).

### 2.2. Serosurvey Methodology

Of the 130 studies, 93 (71%) used EIAs to estimate IgG seroprevalence, with the most commonly used kits being Enzygnost (Siemens, Marburg, Germany, *n* = 28) and EUROIMMUN (EUROIMMUN, Lubeck, Germany, *n* = 20). In 25 (19%) studies, automated chemiluminescent immunoassay (CLIA), chemiluminescent microparticle immunoassay (CMIA), or enzyme-linked fluorescent assay (ELFA) technologies were utilized, with DiaSorin Liaison or Liaison XL (DiaSorin, Saluggia, Italy, *n* = 15) being the most common. Additionally, Luminex-based MBAs were used in seven of the studies (5%). The remaining studies utilized other methods such as the hemagglutination inhibition test, rapid immunochromatographic test, particle agglutination assay, or the specific method was not indicated. Notably, one study compared the performance of the Enzygnost and EUROIMMUN IgG EIAs in a serosurvey of rubella antibodies in pregnant women [66]. In that study, the authors observed a strong qualitative (96.3%) and quantitative (mean titer difference: 0.8 IU/mL) agreement between the two tests, indicating that results between certain EIA tests may be comparable. However, the sensitivity of certain EIA kits may still cause underestimates of seroprevalence. A study in Singapore using the Enzygnost EIA as the primary test method performed further testing on all samples that were negative for measles-specific IgG and a random sampling of equivocal specimens for additional testing. Subsequent testing by PRNT revealed that approximately 75% of the samples initially categorized as measles IgG-negative, along with all randomly selected samples categorized as measles IgG-equivocal, were actually seropositive. This had a significant impact on the estimated measles seroprevalence [67].

Sample sizes varied greatly across the 130 studies, ranging from sample sizes as small as 20 persons to as large as 32,502 individuals. In some instances, the seroprevalence was only reported for specific subgroups and not for the entire study population. As a result, some studies reported seroprevalence estimates for a very small number of people, with 24 (18%) studies reporting seroprevalence for groups of <200 individuals. Overall, the median sample population size for all studies was 561, and 63 (48%) studies surveyed populations of at least 1000 individuals (Figure 1B). Only 37 (28%) studies described the use of a priori sample size analysis to determine the appropriate sample size, as recommended by the WHO Guidelines for the assessment of measles and rubella seroprevalence. Other studies (*n* = 39) described the use of sampling methods, such as multistage random cluster sampling. Most studies (*n* = 92) did not specify a specific statistical method for participant selection. Instead, samples in those studies were obtained from residual blood donor or patient serum specimens, as well as from pre-employment or prenatal samples. Alternatively, many studies that actively recruited participants used targeted enrollment strategies, such as all students enrolled in a particular university program. The use of such opportunistic sampling is common due to its convenience and can provide local data on seroprevalence but cannot be generalized to an entire population.

### 2.3. Serosurvey Demographics

**Figure 2:**
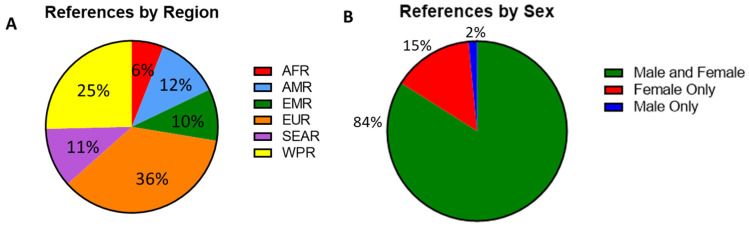
(**A**) Percent of serosurvey studies by region, percentages indicating the proportion of all included studies. African region (AFR, *n* = 8), American region (AMR, *n* = 16), Eastern Mediterranean region (EMR, *n* = 13), European region (EUR, *n* = 48), Southeast Asian region (SEAR, *n* = 15), and Western Pacific region (WPR, *n* = 34). (**B**) A breakdown of gender distribution among participants with percentages indicating the proportion of all included studies. Male and female (*n* = 110), female only (*n* = 19) and male only (*n* = 2).

**Figure 3:**
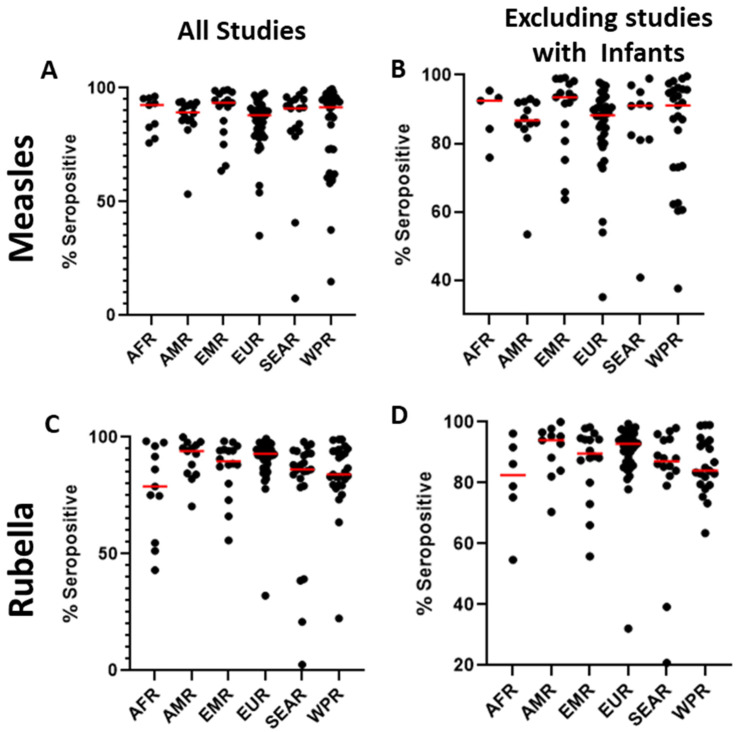
Distribution of seroprevalence for measles (**A**) and rubella (**C**) for all studies included in the literature review and the distribution of seroprevalence for measles (**B**) and rubella (**D**) excluding studies that included infants. Red line represents the median.

The serosurveys included a total of 59 countries; countries in the European WHO region (EUR) were most represented (*n* = 48), followed by the Western Pacific region (WPR, *n* = 34). In contrast, only eight studies reported data from the African region (AFR) (Figure 2A). Among the WHO regions represented in the included studies, the median measles seropositivity ranged from 88% to 93.5% (Table 1, Figure 3A). However, these estimates are certainly influenced by limited sampling in certain regions and differences among the populations studied. In particular, studies including infants at birth or early in life might have skewed the data because of waning maternal antibodies or a lack of exposure in the infants. When studies including infants were not considered, the median measles seropositivity ranged from 86.8% to 93.5% (Table 2, Figure 3B). For rubella, the median seropositivity ranged from 78.9% to 94% (Table 1, Figure 3C). When excluding studies with infants, the range is 82.6% to 94% (Table 2, Figure 3D).

Twelve studies described seroprevalence in asylum seekers, refugees, international adoptees, or other immigrants or displaced peoples, and eleven of those studies examined the migration of persons into European countries from Africa, Asia, or elsewhere in Europe. Within those studies, the median seroprevalence reported in migrants into Europe (79.4% for measles and 85.1% for rubella) was lower than the median seroprevalence reported in other studies of non-migrants in the region (88.5% for measles and 93.5% for rubella). Only 13 surveys were conducted in countries listed by the UN as Least Developed Countries (LDC), and four of those studies were conducted in Zambia. Eighty-three (63%) studies were conducted in developed countries (UN Human Development index >0.8, 2021)—a significant percent increase compared to what was reported by Dimech and Mulders in 2016 [65]. Developed countries have a need to identify immunization gaps and document sufficient levels of population immunity as countries move towards elimination status.

Among the studies, 114 (87%) included adults (aged ≥18 years), 62 (47%) included adolescents (aged 15–17 years), 49 (37%) included children (aged 1–14 years), and 24 (18%) included infants (aged <1 year). Additionally, 110 (84%) of the studies included both male and female subjects. Only two studies (1%) exclusively included men, as these were cohorts of military recruits and professional sports players. Nineteen studies (15%) included only women. These studies focused on either pregnant women, women of childbearing age (WCBA), or women in universities or other workplaces (Figure 2B).

### 2.4. Overall Seroprevalence and Antibody Titers

The median measles seroprevalence reported in all studies was 89.87%, with a wide range of 7.5–99.6%. Only 16 (15%) of measles studies reported quantitative test results. Among those, the median average titer reported was 1153 mIU/mL (range = 77–25,300 mIU/mL). The median rubella seroprevalence reported was 90.5% (range = 2.5–100%). Only 17 rubella studies (17%) reported quantitative test results. Of those, the median average titer reported was 51 IU/mL (range = 4–240 IU/mL). The wide range of reported seroprevalence and antibody titers are significantly influenced by studies in older infants, which is discussed in further detail below. For this reason, the median seroprevalence (as opposed to the mean) likely gives a more accurate estimation of average seroprevalence and antibody titers globally and by region.

### 2.5. Age and Seroprevalence

Because the measles antibody is long lasting, seroprevalence in a particular birth cohort typically continues to increase as the cohort ages and experiences accumulated disease or vaccine-induced immunity. However, different birth cohorts experience different disease and immunization rates, and relative troughs in seroprevalence may be seen in some older cohorts. To assist in identifying immunity gaps, analysis of these patterns of seroprevalence is a major reason for serosurveys. In studies that analyzed age as a variable among adults, adolescents, and children younger than one year (*n* = 78), researchers found that measles and rubella seroprevalence varied by age. Specifically, 41 studies reported an increase in measles seroprevalence among older adults compared to younger participants, as expected, whereas 22 studies observed no age-related differences. Similarly, 30 studies noted a lower rubella antibody prevalence among younger participants than older ones, but 27 studies reported no clear relationship between age and rubella antibody seroprevalence.

The median measles seroprevalence reported for studies that surveyed only infants was 90%. Among the eight studies that reported measles seroprevalence for infants only, there was a wide range of seropositivity (7.5–98%). This is due to differences in the exact age of infants in the study, and the fact that maternally acquired antibody titers wane between birth and the receipt of the measles-containing vaccine. In the four studies that further divided measles seroprevalence in infants by month of age, they all reported declining seroprevalence over the first year of life, as expected [68,69,70,71]. Differences in the exact age group studied make comparisons between studies difficult. However, there are some notable similarities. Cho et al. found that while measles seroprevalence was 94.4% in Korean infants <1 month old, seroprevalence dropped to 27.3% in 4-month-olds. Almost no infants ≥5 months old tested positive for the measles antibody [68]. Similarly, Muthiah et al. reported seropositivity for measles of 95% in Sri Lankan newborns, 23.1% in 7–8-month-olds, and 0% in 11–12-month-olds [69].

Only two studies examined rubella seroprevalence in infants by months of age, but the same pattern was observed as with measles. Muthiah et al. reported rubella seropositivity of 95% in Sri Lankan newborns, 9.6% in 7–8-month-olds, and 0% in 11–12-month-olds [69]. Similarly, Bassal et al. reported 80.4% rubella seropositivity in Israeli infants aged 0–6 months, compared with only 13% in infants aged 6–11 months [72]. In these studies, although each age group contained relatively few individuals, these data on measles and rubella seroprevalence in infants may cumulatively indicate a large window of susceptibility, which is most concerning for infants living in endemic settings. Whether infants should be vaccinated against measles and rubella earlier than current WHO recommendations (9 months of age in countries where transmission is ongoing) is an area of active debate. However, there is currently insufficient evidence on the long-term efficacy of early vaccination [73]. Given the increases in infant seronegativity seen in many studies, it is critical that infants be protected from exposure to measles by decreasing incidence in older populations, while research is being conducted on the immunogenicity of vaccinating infants at ages earlier than 9 months.

### 2.6. Seroprevalence and Vaccination

Sixty-eight studies had vaccination records for participants or surveyed them as to their recollection of previous vaccinations. In 24 of these, seropositivity for measles and/or rubella was reported to be positively associated with vaccination status or an increasing number of doses. Five studies found no statistically significant relationship or, interestingly, that vaccination status was negatively associated with seropositivity [74,75,76,77,78]. Three studies found higher titers of measles and rubella IgG in non-vaccinated individuals compared to those that had been vaccinated, as has been previously reported for individuals following infection [65,69,79,80,81]. This phenomenon was observed in countries that have had circulating measles or rubella viruses during the lifetime of the study participants.

Several studies reported higher seropositivity estimates for measles or rubella among the population than the percentage of participants reporting vaccination. This can be easily attributed to a prior infection with measles or the rubella virus. However, 10 studies reported lower seropositivity than the percentage of individuals either with vaccination records or those that recalled vaccination or infection [82,83,84,85,86,87,88,89,90,91]. These were typically studies of students or employees with available vaccination records. While such single-site studies can only give information on local conditions, this pattern of lower seroprevalence was observed in multiple countries spanning four WHO regions (AMR, EMR, EUR, and WPR) and may suggest instances of vaccine failure or waning immunity in the populations surveyed.

Ten studies described changes in national vaccination programs over time [80,92,93,94,95,96,97,98,99,100]. The results were mixed as to whether programmatic changes resulted in increased or decreased seropositivity for measles and/or rubella. For example, Gorun et al. surveyed 6914 WCBA who had undergone TORCH screening. When dividing the cohort by year of birth and vaccination program eligibility, they found the lowest rubella seroprevalence rates among the youngest cohort—82.4% of those born from 1997 to 2004 who were eligible for the measles–mumps–rubella (MMR) vaccine through the family practice system. In comparison, the seroprevalence was 95.8% in those born in 1989–1994 who were eligible for the monovalent rubella vaccine distributed in schools [92]. Coppeta et al. found no change in measles seropositivity in students following the implementation of a National Plan of Vaccine Prevention (NPVP) in Italy [100].

The effectiveness of supplementary immunization activities (SIAs) on a national or regional level was assessed in seven studies [101,102,103,104,105,106,107]. Of those studies, six described overall higher measles and/or rubella seroprevalence in eligible populations following a campaign, while one showed inconsistent results. Ichimura et al. evaluated the effectiveness of 2015 and 2019 SIAs in East Sepik, Papua New Guinea, and concluded that the 2019 SIA resulted in a higher measles and rubella seroprevalence in the target age group than the 2015 SIA. However, the authors note that the small number of participants (*n* = 278) was a significant limitation. Studies assessing seroprevalence following changes in the national immunization program or the implementation of SIAs should ideally be significantly powered, have sufficient documentation of vaccination, and be conducted over an appropriate timescale in order to reliably draw definitive conclusions on the impact of these initiatives.

## 3. Discussion

### Future Considerations for Measles and Rubella Serosurveillance

Serosurveillance studies provide useful estimates of population immunity and the impact of vaccination programs, which can subsequently inform country-specific priorities and public health initiatives. Countries that have verified elimination status for measles and/or rubella, as well as countries that are approaching elimination, must document that adequate levels of population immunity are maintained or achieved. Furthermore, it is important for countries to understand the presence of gaps in population immunity, such as specific age groups or vulnerable communities that may be susceptible to measles or rubella outbreaks upon the introduction of disease. However, for accurate population-level estimates to be obtained from serosurveys, studies must have been designed and conducted appropriately.

Studies can benefit from a priori study design, including sample size calculations and a delimitation of the study population to ensure it is representative. Although it is recognized that using residual samples from other studies is a practical means to conduct serosurveillance, the results from these studies have inherent limitations. Residual samples may not be wholly representative of the overall population, preventing the generalization of immunity estimates. Also, the number of residual specimens available for testing may be limited, thereby limiting statistical power and potentially undermining confidence in the resulting immunity estimates. Even studies that incorporate active sampling into their design should carefully balance population demographics and the geographical distribution of clinical sites as best as possible to ensure a representative sample is collected. Only with a robust study design that accounts for these aspects can we confidently extrapolate results to the population of interest.

Globally standardizing testing methods and interpreting results across laboratories pose challenges for both measles and rubella [25,57,108]. As noted in our literature search, only 16 (12%) measles studies and 17 (17%) rubella studies reported a titer enabling quantitative analysis of data between studies. Most of the reviewed studies reported only qualitative data, determining the seropositive/seronegative status of samples. Comparison of qualitative data is further complicated by use of multiple different EIA platforms, which may be calibrated differently. Most commercial EIAs are designed for single patient testing, not population-level analyses, and thus have relatively high assay cutoffs, which can impact test sensitivity. Antibody titers following vaccination against measles and rubella are generally lower than following infection [109,110], which may lead to an underestimation of seroprevalence in a vaccinated population if a test with insufficient sensitivity is used. This may lead to the unnecessary implementation of vaccination campaigns or other costly public health measures, highlighting the need for sufficiently sensitive, standardized testing methods for use in serosurveillance.

In a systematic review, MBA was found to have robust sensitivity [56]. In this study, MBA for measles-specific IgG was shown to have a median sensitivity of 95% (compared to PRNT; IQR, 89.8–95.0). This sensitivity was similar to the reported median sensitivity of the Enzygnost EIA (92.1%, IQR 82.3–95.7), a commercial EIA kit that was highly employed in serosurveillance studies but has since been discontinued. Furthermore, MBA has shown a strong correlation to “gold standard” assays for evaluating immunity to measles and rubella. Measles-specific MBA has exhibited a strong correlation with PRNT, which is notable considering that most measles-specific EIAs have a poor correlation with PRNT [64]. In the case of rubella, MBA data were found to have a strong correlation with quantitative ELISA, which is commonly used for the evaluation of humoral immunity against rubella [63].

Multiplex assays, like the MBA, can also be more cost-effective than some commonly used immunoassays as they allow for the simultaneous measurement of antibody concentrations for multiple pathogens using a low sample volume [64]. This is due to the increased sample throughput and the sparing of samples and antigens, in addition to the decrease in human labor [111]. For example, 100 samples can be easily tested simultaneously for measles and rubella IgG on MBA using ~75% fewer resources (e.g., assay plates, reagents) than would be needed to test with single-plex ELISA for both pathogens. MBA also requires significantly less (~10 fold) viral antigen than is needed for conventional ELISA to test an equivalent number of specimens for measles and rubella, further highlighting the cost-savings that can be realized with multiplex testing platforms.

The capability of MBA to measure antibody levels against multiple pathogens simultaneously has additional advantages for informing public health decisions beyond monitoring existing vaccination programs. Serological MBAs can be used to quantify the burden of endemic diseases for which a vaccine has yet to be introduced into the immunization program, enabling public health programs to make informed decisions on the need and strategies for the introduction of new vaccines. Multiplex serology assays also enable programs to better leverage limited resources and strategize coordinated surveys for multiple pathogens [112]. For example, blood samples are routinely collected for different programs such as demographic and health surveys, transmission assessment surveys, and indicator surveys for HIV [23]. Serosurveillance for measles, rubella, or other pathogens could be readily integrated with these programs using MBA to maximize the use of the collected samples and related resources.

MBA has been successfully employed by the Centers for Disease Control and Prevention (CDC) for measles and rubella serosurveillance in collaboration with numerous countries and international non-profit organizations. Recently, we assessed over 2500 dried blood spots (DBS) samples from the Democratic Republic of Congo to quantify measles and rubella seroprevalence in parallel with screening of diphtheria and tetanus antibodies [113]. As the Democratic Republic of Congo has not yet implemented rubella vaccination, this illustrates the utility of MBA to simultaneously enable monitoring of vaccination programs (measles, tetanus, diphtheria), while assessing endemic disease burden (rubella) in the same survey.

MBA is amenable to technology transfer between laboratories, enabling multiplex-based serosurveillance to be conducted in multiple countries. As part of a partnership initiative for strengthening integrated serosurveillance, the CDC and the Pan-American Health Organization (PAHO) established a collaborative effort with delegates from Brazil, Mexico, and Paraguay to pilot the introduction of the MBA in their respective countries as a means of monitoring population immunity and disease transmission for multiple diseases, including measles and rubella [114]. This initiative is intended to showcase the utility of integrated serosurveillance using the MBA, combining surveillance programs that are usually addressed separately but overlap within the same populations and geographic areas. As with any serosurvey, linking the results to vaccination records and historical reports of cases and outbreaks will be critical.

## 4. Conclusions

Serosurveys remain useful tools for supporting elimination efforts for measles and rubella, but only if implemented with the appropriate design considerations and utilizing sufficiently sensitive assays to generate meaningful estimates of population immunity. Complementing overall seroprevalence data with seroprevalence estimates stratified across relevant demographic groups, such as age cohorts and geographic areas, is key for targeting interventions and assessments of program impacts. Unfortunately, this granular approach can be challenging and costly to ensure that there is sufficient statistical power across different strata. The number of published serosurveillance studies detailing measles and rubella immunity estimates has steadily increased over the past decade, yet studies largely continue to implement sub-optimal survey designs using a variety of serological assays. These limitations were previously noted by Dimech and Mulders nearly a decade ago [65,115], and we reiterate the need here for updated, standardized guidelines for conducting seroprevalence studies in support of measles and rubella elimination. Moreover, a standardized serological assay with high sensitivity—such as the MBA—will be critical for accurately assessing population immunity for measles and rubella as more countries achieve elimination.

## Figures and Tables

**Table 1:** Overall median seropositivity by WHO region for measles or rubella based on studies included in this review.

Region	Measles	Rubella
AFR	92.5%	78.9%
AMR	89.3%	94%
EMR	93.5%	89.7%
EUR	88%	92.9%
SEAR	91%	86.2%
WPR	91.5%	86%

**Table 2:** Median seropositivity by WHO region for measles or rubella based on studies included in this review, excluding studies with data from infants.

Region	Measles	Rubella
AFR	92.5%	82.6%
AMR	86.8%	94%
EMR	93.5%	89.7%
EUR	88.3%	92.8%
SEAR	91%	87.1%
WPR	91.1%	84%

## Data Availability

No new data were created or analyzed in this study. Data sharing is not applicable to this article.

## Supplementary Materials

The following supporting information can be downloaded at https://www.mdpi.com/article/10.3390/vaccines12070816/s1, Supplementary Table S1: List of study populations of measles serosurveys included in literature review and Supplementary Table S2: List of study populations of rubella serosurveys included in literature review.
