# Supplementary material for: Serosurveillance for Measles and Rubella
Source: Vaccines (Basel). 2024 Jul 22;12(7):816. doi: 10.3390/vaccines12070816 (PMC11281569; doi:10.3390/vaccines12070816)
Supplement: Supplementary file 1 [file vaccines-12-00816-s001.zip › vaccines-3050654-supplementary.pdf]

**Supplementary Table S1.** List of study populations of measles serosurveys included in literature review.

| Author | Reference | Year | Study group* | Region† | Gender | Age group | Sample size | GMT (mIU/mL)‡ | Final prevalence (%) | 95% CI§ | Method¶ |
|---|---|---|---|---|---|---|---|---|---|---|---|
| Kanamori, et al. | [1] | 2014 | Workplace | WPR | Both | Adults | 243 | NA | 87.2 | NA | EIA |
| Kanamori, et al. | [1] | 2014 | Workplace | WPR | Both | Adults | 2664 | NA | 95.5 | NA | EIA |
| Black, et al. | [2] | 2015 | Workplace | AFR | Both | Adolescents and adults | 1011 | NA | 95.4 | NA | EIA |
| Garcia-Comas, et al. | [3] | 2015 | Population study | EUR | Both | Children to adults | 4380 | NA | 97.8 | 97.3-98.2 | EIA |
| Izadi, et al. | [4] | 2015 | Minors | EMR | Both | Children | 663 | NA | 94.6 | NA | EIA |
| Lebo, et al. | [5] | 2015 | Population study | AMR | Both | Children to adults | 5054 | NA | 92 | 90.9-93.0 | EIA |
| Levine, et al. | [6] | 2015 | Population study | EUR | Boths | Adults | 439 | NA | 85.7 | NA | ELFA |
| Levine, et al. | [6] | 2015 | Population study | EUR | Boths | Adults | 439 | NA | 95.6 | NA | ELFA |
| Lewis, et al. | [7] | 2015 | Workplace | AMR | Both | Adolescents and adults | 32502 | NA | 81.6 | 81.2, 82.0 | Luminex |
| Mao, et al. | [8] | 2015 | WCBA | WPR | Female | Adolescents and adults | 2057 | NA | 95.9 | 93·9–97·3 | EIA |
| Plans, et al. | [9] | 2015 | Minors | EUR | Both | Infants | 353 | 25300 | 90 | NA | EIA |
| Plans, et al. | [9] | 2015 | Pregnant women | EUR | Female | Adolescents and adults | 353 | NA | 89 | NA | EIA |
| Rosario-Rosario, et al. | [10] | 2015 | Population study | AMR | Both | Adults | 460 | NA | 85.8 | NA | EIA |
| Trevisan, et al. | [11] | 2015 | Workplace | EUR | Both | Adults | 4195 | NA | 84.8 | NA | EIA |
| Al-Mekaini, et al. | [12] | 2016 | Minors | EMR | Both | Children | 227 | NA | 98.2 | 95.5, 99.5 | EIA |
| Andrew, et al. | [13] | 2016 | Workplace | WPR | Both | Adults | 1901 | NA | 88 | NA | CLIA |
| Bakri, et al. | [14] | 2016 | Workplace | WPR | Both | Adults | 493 | NA | 75.2 | NA | EIA |
| Cho, et al. | [15] | 2016 | WCBA | WPR | Female | Adults | 80 | NA | 96.3 | NA | EIA |
| Cho, et al. | [15] | 2016 | Minors | WPR | Both | Infants | 295 | NA | 14.9 | NA | EIA |
| Gohil, et al. | [16] | 2016 | Population study | SEAR | Both | Adults | 192 | NA | 91 | 87–95 | EIA |
| Guanche Garcell, et al. | [17] | 2016 | Workplace | EMR | Both | Adults | 705 | NA | 85.6 | 82.4 to 88.4 | EIA |
| Hazlina, et al. | [18] | 2016 | Population study | WPR | Both | Children to adults | 1541 | NA | 87 | 85-89 | EIA |
| Jablonka, et al. | [19] | 2016 | Population study | EUR | Both | Children to adults | 678 | NA | 92.6 | NA | EIA |
| Kader, et al. | [20] | 2016 | Workplace | EUR | Both | Adolescents and adults | 180 | NA | 82.8 | NA | EIA |
| Tafuri, et al. | [21] | 2016 | Population study | EUR | Both | Adults | 1764 | NA | 95.1 | 94.1-96.1 | CLIA |
| Takemoto, et al. | [22] | 2016 | Minors | WPR | Both | Infants | 378 | NA | 98 | NA | EIA |
| Takemoto, et al. | [22] | 2016 | Minors | WPR | Both | Infants | 561 | NA | 95 | NA | EIA |
| Toikkanen, et al. | [23] | 2016 | Population study | EUR | Both | Children to adults | 23647 | NA | 79.9 | 79.4–80.4 | EIA |
| Gioula, et al. | [24] | 2017 | Population study | EUR | Both | Infants to adults | 611 | NA | 82.07 | NA | EIA |
| Jablonka, et al. | [25] | 2017 | Population study | EUR | Both | Children and adults | 552 | NA | 88.5 | NA | EIA |
| Kader, et al. | [26] | 2017 | Pregnant women | EUR | Female | Adults | 176 | NA | 97.2 | NA | EIA |
| Koivisto, et al. | [27] | 2017 | Workplace | EUR | Both | Adults | 157 | NA | 81.5 | 75.4–87.6 | EIA |

| | | | | | | | | | | | |
|---|---|---|---|---|---|---|---|---|---|---|---|
| Sutcliffe, et al. | [28] | 2017 | Minors | AFR | Both | Children and adolescents | 617 | 2606 | 92.5 | 90.4, 94.8 | EIA |
| Wang, et al. | [29] | 2017 | Minors | WPR | Both | Children | 755 | NA | 91.13 | 89.52-92.83 | EIA |
| Conway, et al. | [30] | 2018 | Workplace | AMR | Male | Adults | 98 | NA | 89.7 | | EIA |
| Freidl, et al. | [31] | 2018 | Population study | EUR | Both | Adults | 622 | NA | 88 | 83-93 | Luminex |
| Hachiya, et al. | [32] | 2018 | Population study | WPR | Both | Children to adults | 2135 | NA | 83.9 | 83.8-84 | EIA |
| Hubschen, et al. | [33] | 2018 | Population study | EUR | Both | Adolescents and adults | 406 | NA | 72.7 | NA | EIA |
| Izadi, et al. | [34] | 2018 | Population study | EMR | Both | Adolescents and adults | 253 | NA | 91.7 | 88.3-95.1 | EIA |
| Izadi, et al. | [34] | 2018 | Population study | EMR | Both | Children and adolescents | 265 | NA | 97.4 | 95.4-99.3 | EIA |
| Izadi, et al. | [34] | 2018 | Minors | EMR | Both | Children | 265 | NA | 98.9 | 97.6-100 | EIA |
| Izadi, et al. | [34] | 2018 | Minors | EMR | Both | Children | 273 | NA | 98.9 | 97.6-100 | EIA |
| Meng, et al. | [35] | 2018 | Infants | WPR | Both | Infants | 194 | NA | 59.3 | 52.2-65.9 | EIA |
| Meng, et al. | [35] | 2018 | Pregnant women | WPR | Female | Adults | 194 | NA | 60.3 | 53.3-66.9 | EIA |
| Murray, et al. | [36] | 2018 | Pregnant women | AMR | Female | Adults | 49 | NA | 92 | NA | EIA |
| Murray, et al. | [36] | 2018 | Infants | AMR | Both | Infants | 49 | NA | 94 | NA | EIA |
| Murray, et al. | [36] | 2018 | Pregnant women | SEAR | Female | Adults | 258 | NA | 95 | NA | EIA |
| Murray, et al. | [36] | 2018 | Infants | SEAR | Both | Infants | 258 | NA | 96 | NA | EIA |
| Scepanovic, et al. | [37] | 2018 | Population study | EUR | Both | Adults | 1000 | NA | 91 | NA | Multiplex Flow Immunoassay |
| Shoho, et al. | [38] | 2018 | Workplace | WPR | Female | Adults | 841 | NA | 99.6 | NA | Particle agglutination assay |
| Tomaskova, et al. | [39] | 2018 | Population study | EUR | Both | Children to adults | 3111 | NA | 93 | 92-93.9 | EIA |
| Antona, et al. | [40] | 2019 | Population study | EUR | Both | Adults | 4643 | NA | 90.8 | 89.0-92.1 | EIA |
| Coppeta, et al. | [41] | 2019 | Workplace | EUR | Both | Adults | 319 | NA | 73.7 | NA | CLIA |
| Gupta, et al. | [42] | 2019 | Minors | SEAR | Both | Children | 196 | 1130 | 40.8 | NA | EIA |
| Hagstam, et al. | [43] | 2019 | Pregnant women | EUR | Female | Adults | 940 | NA | 78.93 | NA | EIA |
| Hagstam, et al. | [43] | 2019 | Population study | EUR | Both | Adults | 969 | NA | 90.2 | NA | EIA |
| Hayford, et al. | [44] | 2019 | Population study | AFR | Both | Infants to adults | 590 | NA | 95.5 | 92.8-97.2 | EIA |
| Holka, et al. | [45] | 2019 | Population study | EUR | Both | Infants to adults | 363 | NA | 78.02 | 73.48-81.98 | CLIA |
| Jung, et al. | [46] | 2019 | Workplace | WPR | Both | Adults | 7411 | NA | 73 | 72-74 | EIA |
| Khetsuriani, et al. | [47] | 2019 | Population study | EUR | Both | Adults | 3125 | NA | 93.7 | 92.4-95.1 | EIA |
| Marchi, et al. | [48] | 2019 | Pregnant women | EUR | Female | Adults | 193 | 1409 | 96.9 | 93.3-98.8 | EIA |
| Odemis, et al. | [49] | 2019 | Workplace | EUR | Both | Adolescents and adults | 326 | NA | 74.9 | 70.2-79.6 | EIA |
| Rasheed, et al. | [50] | 2019 | Workplace | AMR | Both | Adults | 71 | NA | 93 | NA | EIA |
| Ristic, et al. | [51] | 2019 | Population study | EUR | Both | Children to adults | 3199 | NA | 86.9 | 85.68-88.05 | CLIA |
| Santosh, et al. | [52] | 2019 | Population study | SEAR | Both | Adults | 335 | NA | 81.16 | NA | ELFA |
| Staehelin, et al. | [53] | 2019 | Population study | AFR | Both | Adolescents and adults | 133 | 639 | 75.9 | NA | EIA |

| | | | | | | | | | | | |
|---|---|---|---|---|---|---|---|---|---|---|---|
| Vos, et al. | [54] | 2019 | Population study | AMR | Both | Infants to adults | 1129 | 928 | 93.7 | 91.9-95.4 | Luminex |
| Vos, et al. | [55] | 2019 | Population study | AMR | Both | Infants to adults | 1829 | 930 | 93.8 | 92.3-95.2 | Luminex |
| Wangchuk, et al. | [56] | 2019 | Population study | SEAR | Both | Children to adults | 1325 | NA | 81 | 78-85 | EIA |
| Wanlapakorn, et al. | [57] | 2019 | Population study | SEAR | Both | Infants to adults | 1781 | 653 | 84.3 | 82.6-86 | EIA |
| Adam, et al. | [58] | 2020 | Population study | EMR | Both | Adults | 153 | 4176 | 93.5 | NA | EIA |
| Adam, et al. | [58] | 2020 | Workplace | EMR | Both | Adults | 239 | 6234 | 99.2 | NA | EIA |
| Adam, et al. | [58] | 2020 | Minors | EMR | Both | Children and adolescents | 294 | 1280 | 93.5 | NA | EIA |
| Bechini, et al. | [59] | 2020 | Minors | EUR | Both | Children and adolescents | 1870 | NA | 35.1 | NA | CLIA |
| Borocz, et al. | [60] | 2020 | Population study | EUR | Both | Children to adults | 3523 | NA | 89.84 | NA | EIA |
| Breakwell, et al. | [61] | 2020 | Minors | WPR | Both | Children | 1156 | NA | 99 | 98-99 | Luminex |
| Estofolete, et al. | [62] | 2020 | Population study | AMR | Both | Children to adults | 981 | NA | 84.2 | NA | EIA |
| Feldstein, et al. | [63] | 2020 | Minors | SEAR | Both | Children | 273 | NA | 97 | 94-99 | Luminex |
| Feldstein, et al. | [63] | 2020 | Minors | SEAR | Both | Children and adolescents | 299 | NA | 99 | 96-100 | Luminex |
| Feldstein, et al. | [63] | 2020 | Minors | SEAR | Both | Children | 358 | NA | 91 | 86-94 | Luminex |
| Karadeniz, et al. | [64] | 2020 | Workplace | EUR | Both | Adults | 1053 | NA | 57.1 | NA | EIA |
| Kwak, et al. | [65] | 2020 | Workplace | WPR | Both | Adults | 2830 | NA | 93.1 | NA | CLIA |
| Lucca, et al. | [66] | 2020 | Workplace | AMR | Both | Adolescents and adults | 12349 | NA | 85.7 | NA | ELFA |
| Madi, et al. | [67] | 2020 | Population study | EMR | Both | Children to adults | 1000 | 4900 | 94.8 | 96.2-93.4 | EIA |
| Maltezou, et al. | [68] | 2020 | Workplace | EUR | Male | Adults | 385 | NA | 80 | NA | EIA |
| Minta, et al. | [69] | 2020 | Minors | AMR | Both | Children | 1146 | NA | 87.4 | 85.1–89.3 | Luminex |
| Nakladalova, et al. | [70] | 2020 | Workplace | EUR | Both | Adults | 3027 | NA | 54 | NA | EIA |
| Ng, et al. | [71] | 2020 | Population study | WPR | Both | Children and adolescents | 1200 | NA | 98.2 | 97.2-98.8 | EIA |
| Nogareda, et al. | [72] | 2020 | Population study | WPR | Both | Infants to adults | 4598 | NA | 94 | 93-95 | EIA |
| Ogawa, et al. | [73] | 2020 | Workplace | WPR | Both | Adults | 2371 | NA | 62.2 | NA | EIA |
| Zahraei, et al. | [74] | 2020 | Population study | EMR | Female | Adolescents and adults | 1569 | NA | 80.7 | 78.7-82.6 | EIA |
| Zanella, et al. | [75] | 2020 | Minors | EUR | Both | Children and adolescents | 165 | NA | 88.5 | NA | EIA |
| Bianchi, et al. | [76] | 2021 | Population study | EUR | Both | Adults | 611 | NA | 84.6 | 81.5-87.4 | CLIA |
| Carcelen, et al. | [77] | 2021 | Minors | AFR | Both | Adolescents and adults | 185 | NA | 93.3 | 84.5-97.3 | EIA |
| Carcelen, et al. | [77] | 2021 | Minors | AFR | Both | Infants and children | 358 | NA | 96.4 | 91.7-98.5 | EIA |
| Carcelen, et al. | [77] | 2021 | Minors | AFR | Both | Adolescents and adults | 540 | NA | 84.3 | 77.8-89.2 | EIA |
| Carcelen, et al. | [77] | 2021 | Minors | AFR | Both | Infants and children | 565 | NA | 77.8 | 73.2-81.9 | EIA |
| Coppeta, et al. | [78] | 2021 | Workplace | EUR | Both | Adults | 1017 | NA | 88 | NA | CLIA |
| Frau, et al. | [79] | 2021 | Workplace | EUR | Female | Adults | 263 | NA | 90.5 | 87.0-94.0 | CLIA |
| Friedrich, et al. | [80] | 2021 | Population study | EUR | Both | Adults | 6802 | NA | 89.9 | 88.6–91.1 | EIA |

| | | | | | | | | | | | |
|---|---|---|---|---|---|---|---|---|---|---|---|
| Gupta, et al. | [81] | 2021 | Minors | SEAR | Both | Infants and children | 50 | NA | 94 | NA | EIA |
| Khampanisong, et al. | [82] | 2021 | Minors | WPR | Both | Infants | 508 | 773 | 58.1 | NA | EIA |
| Khampanisong, et al. | [82] | 2021 | WCBA | WPR | Female | Adolescents and adults | 508 | 4024 | 95.7 | NA | EIA |
| Kim, et al. | [83] | 2021 | Workplace | WPR | Both | Adults | 2885 | NA | 91.9 | NA | CLIA |
| Kostinov, et al. | [84] | 2021 | Workplace | EUR | Both | Adults | 1742 | 1200 | 85 | NA | EIA |
| Mahallawi, et al. | [85] | 2021 | Population study | EMR | Both | Adults | 443 | NA | 92 | NA | EIA |
| Muthiah, et al. | [86] | 2021 | Minors | SEAR | Both | Infants | 280 | 77 | 7.5 | NA | EIA |
| Muthiah, et al. | [86] | 2021 | WCBA | SEAR | Female | Adolescents and adults | 294 | 996 | 91.5 | NA | EIA |
| Muthiah, et al. | [86] | 2021 | Minors | SEAR | Both | Infants | 294 | 1176 | 95 | NA | EIA |
| Norman, et al. | [87] | 2021 | Population study | EUR | Both | Infants to adults | 468 | NA | 78.4 | NA | NA |
| von Linstow, et al. | [88] | 2021 | Workplace | EUR | Both | Adults | 555 | NA | 90.3 | NA | CLIA |
| Xaydalasouk, et al. | [89] | 2021 | Population study | WPR | Both | Children to adults | 2463 | NA | 73.4 | NA | EIA |
| Yang, et al. | [90] | 2021 | Minors | WPR | Both | Infants and children | 938 | 1210 | 94 | 92.5–95.5 | EIA |
| Yoo, et al. | [91] | 2021 | Workplace | WPR | Female | Adults | 587 | NA | 93.9 | NA | EIA |
| Carcelen, et al. | [92] | 2022 | Population study | AFR | Both | Infants to adults | 9854 | NA | 82.8 | 81.6-83.9 | EIA |
| Hase, et al. | [93] | 2022 | Workplace | WPR | Both | Adults | 85 | NA | 37.6 | NA | EIA |
| Ichimura, et al. | [94] | 2022 | Population study | WPR | Both | Children to adults | 278 | NA | 62.6 | 56.6–68.3 | EIA |
| Lin, et al. | [95] | 2022 | Workplace | WPR | Both | Adults | 2905 | NA | 73 | NA | CLIA |
| Murhekar, et al. | [96] | 2022 | Minors | SEAR | Both | Infants to adolescents | 2570 | NA | 78.8 | NA | EIA |
| Murhekar, et al. | [96] | 2022 | Minors | SEAR | Both | Infants to adolescents | 2619 | NA | 92.1 | NA | EIA |
| Nokhodian, et al. | [97] | 2022 | Minors | EMR | Both | Children and adolescents | 1678 | NA | 65.8 | NA | EIA |
| Quach, et al. | [98] | 2022 | Population study | AMR | Both | Adults | 1393 | 158 | 53.4 | NA | EIA |
| Virachith, et al. | [99] | 2022 | Population study | WPR | Both | Children to adults | 363 | NA | 60.6 | NA | EIA |
| Chung, et al. | [100] | 2023 | Workplace | WPR | Both | Adults | 3173 | NA | 94.8 | NA | EIA |
| Gusmao, et al. | [101] | 2023 | Workplace | SEAR | Both | Adolescents and adults | 324 | NA | 82.4 | 77.8–86.4 | EIA |
| Kia, et al. | [102] | 2023 | Population study | EMR | Both | Adults | 450 | NA | 63.6 | NA | EIA |
| Miyano, et al. | [103] | 2023 | Population study | WPR | Both | Children to adults | 2001 | NA | 97.5 | 96.7-98.0 | EIA |
| Pedranti, et al. | [104] | 2023 | Minors | AMR | Both | Children and adolescents | 180 | NA | 92.2 | NA | ELFA |
| Santacruz-Sanmartin, et al. | [105] | 2023 | Minors | AMR | Both | Infants | 734 | 662 | 88.9 | NA | EIA |
| Santacruz-Sanmartin, et al. | [105] | 2023 | Pregnant women | AMR | Female | Adolescents and adults | 790 | 552 | 86.1 | NA | EIA |

Footnotes:
* Study group: WCBA = women of childbearing age
† Region: AFR = African Region, AMR = Region of the Americas, EUR = European Region, EMR = Eastern Mediterranean Region, SEAR = South-East Asian Region, WPR = Western Pacific Region
‡ GMT (mIU/mL): NA = Not applicable, as assay used was a qualitative assay
§ 95% CI: NA = Not available, data was not reported in the study
¶ Method: CLIA = chemiluminescent immunoassay, EIA = Enzyme Immunoassay, ELFA = enzyme-linked fluorescence assay, NA= not available

**Supplementary Table S2.** List of study populations of rubella serosurveys included in literature review.

| Author | Reference | Year | Study group* | Region† | Gender | Age group | Sample size | GMT (IU/mL)‡ | Final prevalence (%) | 95% CI§ | Method¶ |
|---|---|---|---|---|---|---|---|---|---|---|---|
| Gadallah, et al. | [106] | 2014 | WCBA | EMR | Female | Adults | 339 | NA | 88.2 | 84.8–91.6 | EIA |
| Lo Giudice, et al. | [107] | 2014 | Population study | EUR | Female | Adults | 489 | NA | 82.2 | 78.8–85.6 | CLIA |
| Black, et al. | [2] | 2015 | Workplace | AFR | Both | Adolescnets to adults | 919 | NA | 86.2 | NA | EIA |
| Chua, et al. | [108] | 2015 | Population study | WPR | Both | Adults | 3293 | NA | 85 | 83.7–86.2 | CLIA |
| Chua, et al. | [108] | 2015 | Population study | WPR | Both | Adults | 4153 | NA | 84 | 82.9–85.1 | CLIA |
| Garcia-Comas, et al. | [3] | 2015 | Population study | EUR | Both | Children to adults | 4380 | NA | 97.2 | 96.5-97.7 | EIA |
| Lebo, et al. | [5] | 2015 | Population study | AMR | Both | Children to adults | 5054 | NA | 95.3 | 94.3-96.2 | EIA |
| Lewis, et al. | [7] | 2015 | Workplace | AMR | Both | Adolescents and adults | 32502 | NA | 82.1 | 81.7, 82.5 | Luminex |
| Mao, et al. | [8] | 2015 | WCBA | WPR | Female | Adolescents and adults | 1602 | NA | 73.3 | 70·5–76·1 | EIA |
| Plans, et al. | [9] | 2015 | Pregnant women | EUR | Female | Adolescents and adults | 353 | NA | 95 | NA | EIA |
| Plans, et al. | [9] | 2015 | Minors | EUR | Both | Infants | 353 | 5.3 | 96 | NA | EIA |
| Rosario-Rosario, et al. | [10] | 2015 | Population study | AMR | Both | Adults | 460 | NA | 96.6 | NA | EIA |
| Al-Mekaini, et al. | [12] | 2016 | Minors | EMR | Both | Children | 227 | NA | 98.3 | 95.5, 99.5 | EIA |
| Andrew, et al. | [13] | 2016 | Workplace | WPR | Both | Adults | 1901 | NA | 78 | NA | CLIA |
| Bakri, et al. | [14] | 2016 | Workplace | WPR | Boths | Adults | 493 | NA | 88.2 | NA | EIA |
| Cho, et al. | [15] | 2016 | WCBA | WPR | Female | Adults | 80 | NA | 98.8 | NA | EIA |
| Cho, et al. | [15] | 2016 | Minors | WPR | Both | Infants | 295 | NA | 22.4 | NA | EIA |
| Gohil, et al. | [16] | 2016 | Population study | SEAR | Both | Adults | 192 | NA | 88 | 80–96 | EIA |
| Guanche Garcell, et al. | [17] | 2016 | Workplace | EMR | Both | Adults | 705 | NA | 94.7 | 92.2-97.3 | CMIA |
| Jablonka, et al. | [19] | 2016 | Population study | EUR | Both | Children to adults | 678 | NA | 97.8 | NA | CLIA |
| Kader, et al. | [20] | 2016 | Workplace | EUR | Both | Adolescents and adults | 180 | NA | 98.3 | NA | EIA |
| Takemoto, et al. | [22] | 2016 | Minors | WPR | Both | Infants | 378 | NA | 96.3 | NA | EIA |
| Takemoto, et al. | [22] | 2016 | Minors | WPR | Both | Infants | 561 | NA | 94 | NA | EIA |
| Toikkanen, et al. | [23] | 2016 | Population study | EUR | Both | Children to adults | 23643 | NA | 85.1 | 84.7–85.6 | EIA |
| Gallone, et al. | [109] | 2017 | Population study | EUR | Both | Adolescents and adults | 1764 | 4 | 86.7 | 85.0-88.2 | CLIA |
| Jablonka, et al. | [25] | 2017 | Population study | EUR | Both | Children and adults | 554 | NA | 77.9 | NA | CLIA |
| Kader, et al. | [26] | 2017 | Pregnant women | EUR | Female | Adults | 176 | NA | 99.4 | NA | EIA |
| Koivisto, et al. | [27] | 2017 | Workplace | EUR | Both | Adults | 157 | NA | 93 | 89.0–97.0 | EIA |
| Nobrega, et al. | [110] | 2017 | Pregnant women | AMR | Female | Adults and adolescents | 87 | NA | 95.4 | NA | CLIA |

| | | | | | | | | | | | |
|---|---|---|---|---|---|---|---|---|---|---|---|
| Sutcliffe, et al. | [28] | 2017 | Minors | AFR | Both | Children and adolescents | 617 | 240 | 54.7 | 50.6, 58.8 | EIA |
| Conway, et al. | [30] | 2018 | Workplace | AMR | Male | Adults | 98 | NA | 70.4 | NA | EIA |
| Edirisuriya, et al. | [111] | 2018 | Population study | WPR | Both | Children to adults | 2729 | NA | 92.1 | 91-93.2 | EIA |
| Freidl, et al. | [31] | 2018 | Population study | EUR | Both | Adults | 622 | NA | 94 | 84-98 | Luminex |
| Hachiya, et al. | [32] | 2018 | Population study | WPR | Both | Children to adults | 2135 | NA | 75.4 | 75.3-75.5 | EIA |
| Hubschen, et al. | [33] | 2018 | Population study | EUR | Both | Adolescents and adults | 406 | NA | 90.4 | NA | EIA |
| Izadi, et al. | [34] | 2018 | Population study | EMR | Both | Adolescents and adults | 253 | NA | 87.4 | 83.2-91.5 | EIA |
| Izadi, et al. | [34] | 2018 | Population study | EMR | Both | Children and adolescents | 265 | NA | 88.7 | 84.8-92.5 | EIA |
| Izadi, et al. | [34] | 2018 | Minors | EMR | Both | Children | 265 | NA | 94.3 | 91.5-97.1 | EIA |
| Izadi, et al. | [34] | 2018 | Minors | EMR | Both | Children | 273 | NA | 96.3 | 94.1-98.6 | EIA |
| Meng, et al. | [35] | 2018 | Pregnant women | WPR | Female | Adults | 194 | NA | 83 | 77.1-87.6 | EIA |
| Meng, et al. | [35] | 2018 | Infants | WPR | Both | Infants | 194 | NA | 83 | 77.1-87.6 | EIA |
| Murray, et al. | [36] | 2018 | Pregnant women | AMR | Female | Adults | 49 | NA | 94 | NA | EIA |
| Murray, et al. | [36] | 2018 | Infants | AMR | Both | Infants | 49 | NA | 98 | NA | EIA |
| Murray, et al. | [36] | 2018 | Infants | SEAR | Both | Infants | 258 | NA | 93 | NA | EIA |
| Murray, et al. | [36] | 2018 | Pregnant women | SEAR | Female | Adults | 258 | NA | 94 | NA | EIA |
| Scepanovic, et al. | [37] | 2018 | Population study | EUR | Both | Adults | 1000 | NA | 94 | NA | Multiplex Flow Immunoassay |
| Shoho, et al. | [38] | 2018 | Workplace | WPR | Female | Adolescents and adults | 841 | NA | 99 | NA | HI |
| Siira, et al. | [112] | 2018 | Workplace | EUR | Both | Adults | 495 | 21 | 84.6 | 81.2-87.6 | EIA |
| Antona, et al. | [40] | 2019 | Population study | EUR | Both | Adults | 4647 | NA | 94.6 | 93.3-95.7 | EIA |
| Crooke, et al. | [113] | 2019 | Population study | AMR | Both | Adults | 1393 | 41 | 97.8 | NA | EIA |
| Gupta, et al. | [42] | 2019 | Minors | SEAR | Both | Children | 196 | 54 | 86.2 | NA | EIA |
| Hagstam, et al. | [43] | 2019 | Population study | EUR | Both | Adults | 935 | NA | 97.1 | NA | CLIA |
| Hagstam, et al. | [43] | 2019 | Pregnant women | EUR | Female | Adults | 984 | NA | 92.7 | NA | CLIA |
| Hayford, et al. | [44] | 2019 | Population study | AFR | Both | Infants to adults | 590 | NA | 97.7 | 96-98.7 | EIA |
| Khetsuriani, et al. | [47] | 2019 | Population study | EUR | Both | Adults | 3125 | NA | 91.4 | 89.9-92.9 | EIA |
| Marchi, et al. | [48] | 2019 | Pregnant and WCBA | EUR | Female | Adolescents and adults | 383 | NA | 84.3 | 80.4-87.6 | EIA |
| Marchi, et al. | [48] | 2019 | WCBA | EUR | Female | Adolescents and adults | 387 | NA | 88.6 | 85.1-91.4 | EIA |
| Odemis, et al. | [49] | 2019 | Workplace | EUR | Both | Adolescents and adults | 326 | NA | 94.5 | 92-97 | EIA |
| Rasheed, et al. | [50] | 2019 | Workplace | AMR | Both | Adults | 71 | NA | 100 | NA | EIA |
| Santosh, et al. | [52] | 2019 | Population study | SEAR | Both | Adults | 335 | NA | 79.1 | NA | ELFA |

| | | | | | | | | | | | |
|---|---|---|---|---|---|---|---|---|---|---|---|
| Staehelin, et al. | [53] | 2019 | Population study | AFR | Female | Adolescents and adults | 20 | 30 | 78.9 | NA | CMIA |
| Viswanathan, et al. | [114] | 2019 | Pregnant women | SEAR | Female | Adults | 435 | NA | 87.1 | NA | CLIA |
| Vos, et al. | [55] | 2019 | Population study | AMR | Both | Infants to adults | 1829 | 31 | 84.5 | 82.4-86.6 | Luminex |
| Wangchuk, et al. | [56] | 2019 | Population study | SEAR | Both | Children to adults | 1325 | NA | 97 | 95-99 | EIA |
| Wanlapakorn, et al. | [57] | 2019 | Population study | SEAR | Both | Infants to adults | 1781 | 39.5 | 78.5 | 76.6-80.4 | EIA |
| Adam, et al. | [58] | 2020 | Population study | EMR | Both | Adults | 153 | 78 | 97.9 | NA | EIA |
| Adam, et al. | [58] | 2020 | Workplace | EMR | Both | Adults | 239 | 83 | 94.1 | NA | EIA |
| Adam, et al. | [58] | 2020 | Population study | EMR | Both | Children and adolescents | 294 | 158 | 55.8 | NA | EIA |
| Bechini, et al. | [59] | 2020 | Minors | EUR | Both | Children and adolescents | 1868 | NA | 32.1 | NA | CLIA |
| Borocz, et al. | [60] | 2020 | Population study | EUR | Both | Children to adults | 1736 | NA | 92.28 | NA | EIA |
| Breakwell, et al. | [61] | 2020 | Minors | WPR | Both | Children | 1156 | NA | 99 | 97-99 | Luminex |
| Coppeta, et al. | [115] | 2020 | Workplace | EUR | Female | Adults | 514 | 50 | 90.3 | 86.0–93.6 | CLIA |
| Estofolete, et al. | [62] | 2020 | Population study | AMR | Both | Children to adults | 981 | NA | 92.9 | NA | EIA |
| Feldstein, et al. | [63] | 2020 | Minors | SEAR | Both | Children | 273 | NA | 98 | 95-99 | Luminex |
| Feldstein, et al. | [63] | 2020 | Minors | SEAR | Both | Children and adolescents | 299 | NA | 96 | 90-98 | Luminex |
| Feldstein, et al. | [63] | 2020 | Minors | SEAR | Both | Children | 358 | NA | 84 | 79-88 | Luminex |
| Karadeniz, et al. | [64] | 2020 | Workplace | EUR | Both | Adults | 1053 | NA | 96.3 | NA | EIA |
| Madi, et al. | [67] | 2020 | Population study | EMR | Both | Children to adults | 1000 | 25.7 | 73 | 70.4-75.9 | EIA |
| Maltezou, et al. | [68] | 2020 | Workplace | EUR | Male | Adults | 385 | NA | 85.7 | NA | CLIA |
| Minta, et al. | [69] | 2020 | Minors | AMR | Both | Children | 1146 | NA | 84 | 80.5–86.9 | Luminex |
| Motaze, et al. | [116] | 2020 | Population study | AFR | Both | Infants to adults | 6057 | NA | 43 | NA | EIA |
| Ng, et al. | [71] | 2020 | Minors | WPR | Both | Children and adolescents | 1200 | NA | 94.8 | 93.4-95.9 | EIA |
| Nogareda, et al. | [72] | 2020 | Population study | WPR | Both | Infants to adults | 4598 | NA | 95 | 94-96 | EIA |
| Ogawa, et al. | [73] | 2020 | Workplace | WPR | Both | Adults | 2371 | NA | 79.3 | NA | HI |
| Patic, et al. | [117] | 2020 | Population study | EUR | Both | Children to adults | 3404 | NA | 92.9 | 92-93.8 | CLIA |
| Shahapur, et al. | [118] | 2020 | WCBA | SEAR | Female | Adolescents and adults | 115 | NA | 20.86 | NA | EIA and rapid ICT |
| Shahapur, et al. | [118] | 2020 | Pregnant women | SEAR | Female | Adolescents and adults | 125 | NA | 39.2 | NA | EIA and rapid ICT |
| Shashank, et al. | [119] | 2020 | Population study | SEAR | Female | Adolescents and adults | 188 | NA | 85.6 | NA | NA |
| Zahraei, et al. | [74] | 2020 | Women | EMR | Female | Adolescents and adults | 1573 | NA | 90.6 | 89.1-92.0 | EIA |
| Carcelen, et al. | [77] | 2021 | Minors | AFR | Both | Infants and children | 358 | NA | 98.3 | 95.5-99.4 | EIA |
| Carcelen, et al. | [77] | 2021 | Population study | AFR | Both | Adults | 408 | NA | 96.2 | 93-98 | EIA |

| | | | | | | | | | | | |
|---|---|---|---|---|---|---|---|---|---|---|---|
| Carcelen, et al. | [77] | 2021 | Minors | AFR | Both | Infants and children | 565 | NA | 51.3 | 45.6-57 | EIA |
| Coppeta, et al. | [78] | 2021 | Workplace | EUR | Both | Adults | 1017 | NA | 90.3 | NA | CLIA |
| Frau, et al. | [79] | 2021 | Workplace | EUR | Female | Adults | 263 | NA | 94.7 | 92.0-97.4 | CLIA |
| Friedrich, et al. | [80] | 2021 | Population study | EUR | Both | Adults | 6811 | NA | 94 | 93.3–94.7 | EIA |
| Gorun, et al. | [120] | 2021 | WCBA | EUR | Female | Adolescents and adults | 1452 | NA | 94.1 | NA | CLIA and CMIA |
| Gorun, et al. | [120] | 2021 | WCBA | EUR | Female | Adolescents and adults | 5462 | NA | 91.4 | NA | CMIA |
| Gupta, et al. | [81] | 2021 | Minors | SEAR | Both | Infants and children | 50 | NA | 86 | NA | EIA |
| Muthiah, et al. | [86] | 2021 | Minors | SEAR | Both | Infants | 280 | 34 | 2.5 | NA | EIA |
| Muthiah, et al. | [86] | 2021 | WCBA | SEAR | Female | Adolescents and adults | 294 | 51 | 88.1 | NA | EIA |
| Muthiah, et al. | [86] | 2021 | Minors | SEAR | Both | Infants | 294 | 67 | 93 | NA | EIA |
| Norman, et al. | [87] | 2021 | Population study | EUR | Both | Infants to adults | 468 | NA | 81.2 | NA | NA |
| Sasaki, et al. | [121] | 2021 | Population study | WPR | Both | Adults | 370 | NA | 94 | NA | EIA |
| Shanmugasundaram, et al. | [122] | 2021 | Pregnant women | SEAR | Female | Adolescents and adults | 1800 | 81 | 82.3 | 80.4-84.0 | EIA |
| Toizumi, et al. | [123] | 2021 | WCBA | WPR | Female | Adults | 2013 | NA | 79.6 | 81.4-77.9 | EIA |
| Toizumi, et al. | [123] | 2021 | Minors | WPR | Both | Infants | 2013 | NA | 79.6 | NA | EIA |
| Trevisan, et al. | [124] | 2021 | Workplace | EUR | Both | Adults | 1404 | 130 | 86.8 | NA | EIA |
| Trevisan, et al. | [124] | 2021 | Population study | EUR | Both | Adults | 3236 | 105 | 97.6 | NA | EIA |
| Trevisan, et al. | [124] | 2021 | Population study | EUR | Both | Adults | 6382 | 63 | 97 | NA | EIA |
| von Linstow, et al. | [88] | 2021 | Workplace | EUR | Both | Adults | 555 | NA | 92.3 | NA | CLIA |
| Xaydalasouk, et al. | [89] | 2021 | Population study | WPR | Both | Children to adults | 2463 | NA | 93 | NA | EIA |
| Yoo, et al. | [91] | 2021 | Workplace | WPR | Female | Adults | 3711 | NA | 83.4 | NA | CMIA |
| Armah, et al. | [125] | 2022 | Pregnant women | AFR | Female | Adults | 145 | NA | 91.7 | NA | EIA |
| Bassal, et al. | [126] | 2022 | Population study | EUR | Both | Infants to adults | 3169 | NA | 95.2 | 94.4–95.9 | EIA |
| Carcelen, et al. | [92] | 2022 | Population study | AFR | Both | Infants to adults | 9854 | NA | 74.9 | 73.7-76.0 | EIA |
| Hase, et al. | [93] | 2022 | Workplace | WPR | Both | Adults | 85 | NA | 63.5 | NA | EIA |
| Ibrahim, et al. | [127] | 2022 | Population study | EMR | Both | Adolescents and adults | 745 | NA | 94.2 | NA | EIA |
| Ichimura, et al. | [94] | 2022 | Population study | WPR | Both | Children to adults | 278 | NA | 82 | 77.0–86.3 | EIA |
| Lavrentieva, et al. | [128] | 2022 | Population study | AFR | Both | Children to adults | 331 | NA | 75.2 | NA | EIA |
| Lavrentieva, et al. | [128] | 2022 | Population study | EUR | Both | Children to adults | 1400 | NA | 86.8 | NA | EIA |
| Lavrentieva, et al. | [128] | 2022 | Population study | EUR | Both | Children to adults | 13511 | NA | 96.9 | NA | EIA |
| Murhekar, et al. | [96] | 2022 | Minors | SEAR | Both | Infants to adolescents | 2570 | NA | 38.5 | NA | EIA |

| | | | | | | | | | | | |
|---|---|---|---|---|---|---|---|---|---|---|---|
| Murhekar, et al. | [96] | 2022 | Minors | SEAR | Both | Infants to adolescents | 2619 | NA | 92.1 | NA | EIA |
| Nokhodian, et al. | [97] | 2022 | Minors | EMR | Both | Children and adolescents | 1678 | NA | 80.1 | NA | EIA |
| Virachith, et al. | [99] | 2022 | Population study | WPR | Both | Children to adults | 363 | NA | 91.2 | NA | EIA |
| Gusmao, et al. | [101] | 2023 | Workplace | SEAR | Both | Adolescents and adults | 324 | NA | 94.4 | 91.4–96.7 | CLIA |
| Hashemi, et al. | [129] | 2023 | Workplace | EMR | Female | Adults | 434 | NA | 66.1 | NA | EIA |
| Miyano, et al. | [103] | 2023 | Population study | WPR | Both | Children to adults | 2001 | NA | 86.8 | 85.2-88.2 | EIA |
| Pedranti, et al. | [104] | 2023 | Population study | AMR | Both | Children and adolescents | 180 | 31 | 88.3 | NA | CMIA |
| Wang, et al. | [130] | 2023 | Minors | WPR | Both | Children | 778 | 58 | 83 | NA | EIA |

Footnotes:
* Study group: WCBA = women of childbearing age
† Region: AFR = African Region, AMR = Region of the Americas, EUR = European Region, EMR = Eastern Mediterranean Region, SEAR = South-East Asian Region, WPR = Western Pacific Region
‡ GMT (mIU/mL): NA = Not applicable, as assay used was a qualitative assay
§ 95% CI: NA = Not available, data was not reported in the study
¶ Method: CLIA = chemiluminescent immunoassay, CMIA = Chemiluminescent microparticle immunoassay, EIA = Enzyme Immunoassay, rapid ICT = rapid immunochromatographic test, ELFA = enzyme-linked fluorescence assay, HI = hemagglutination-inhibition test, NA= not available

**References:**


1. Kanamori, H., et al., *Prevaccination antibody screening and immunization program for healthcare personnel against measles, mumps, rubella, and varicella in a Japanese tertiary care hospital.* Tohoku Journal of Experimental Medicine, 2014. **234**(2): p. 111-6.
2. Black, A.P., et al., *Serosurveillance of vaccine preventable diseases and hepatitis C in healthcare workers from Lao PDR.* PLoS ONE [Electronic Resource], 2015. **10**(4): p. e0123647.
3. Garcia-Comas, L., et al., *Seroprevalence of measles and rubella virus antibodies in the population of the Community of Madrid, 2008-2009.* Journal of Infection and Public Health, 2015. **8**(5): p. 432-40.
4. Izadi, S., T. Mokhtari-Azad, and S.M. Zahraei, *Measles vaccination coverage and seroprevalence of anti-measles antibody in south-east Islamic Republic of Iran.* Eastern Mediterranean Health Journal, 2015. **21**(6): p. 396-402.
5. Lebo, E.J., et al., *Seroprevalence of measles, mumps, rubella and varicella antibodies in the United States population, 2009-2010.* Open Forum Infectious Diseases, 2015. **2**(1): p. ofv006.
6. Levine, H., et al., *Seroprevalence of measles, mumps and rubella among young adults, after 20 years of universal 2-dose MMR vaccination in Israel.* Human vaccines & Immunotherapeutics, 2015. **11**(6): p. 1400-5.
7. Lewis, P.E., et al., *Measles, Mumps, and Rubella Titers in Air Force Recruits: Below Herd Immunity Thresholds?* American Journal of Preventive Medicine, 2015. **49**(5): p. 757-760.
8. Mao, B., et al., *Immunity to polio, measles and rubella in women of child-bearing age and estimated congenital rubella syndrome incidence, Cambodia, 2012.* Epidemiology & Infection, 2015. **143**(9): p. 1858-67.
9. Plans, P., et al., *Prevalence of anti-rubella, anti-measles and anti-mumps IgG antibodies in neonates and pregnant women in Catalonia (Spain) in 2013: susceptibility to measles increased from 2003 to 2013.* European Journal of Clinical Microbiology & Infectious Diseases, 2015. **34**(6): p. 1161-71.
10. Rosario-Rosario, G., et al., *Using Locally Derived Seroprevalence Data on Measles, Mumps, Rubella, and Varicella by Birth Cohort to Determine Risks for Vaccine-Preventable Diseases During International Travel.* Journal of Travel Medicine, 2015. **22**(6): p. 396-402.
11. Trevisan, A., et al., *Prevalence of measles virus-specific IgG antibodies according to vaccination schedule in medical students of Padua University.* Future Virology, 2015. **10(7)**: p. 817-826.
12. Al-Mekaini, L.A., et al., *Seroprevalence of vaccine-preventable diseases among young children in the United Arab Emirates.* International Journal of Infectious Diseases, 2016. **50**: p. 67-71.
13. Andrew, E.C., et al., *Seroprotection to vaccine-preventable diseases among workers at a Victorian tertiary hospital.* Australian & New Zealand Journal of Public Health, 2016. **40**(3): p. 284-9.
14. Bakri, F.G., et al., *Seroprevalence of measles, mumps, rubella, and varicella amongphysicians and nurses in Jordan.* Turkish Journal of Medical Sciences, 2016. **46**(3): p. 614-9.
15. Cho, H.K., et al., *Seroprevalences of Specific IgG Antibodies to Measles, Mumps, and Rubella in Korean Infants.* Journal of Korean Medical Science, 2016. **31**(12): p. 1957-1962.
16. Gohil, D.J., et al., *Seroprevalence of Measles, Mumps, and Rubella Antibodies in College Students in Mumbai, India.* Viral Immunology, 2016. **29**(3): p. 159-63.
17. Guanche Garcell, H., et al., *Seroprotection against Vaccine-Preventable Diseases amongst Health Care Workers in a Community Hospital, Qatar.* International Journal of Occupational & Environmental Medicine, 2016. **7**(4): p. 234-40.
18. Hazlina, Y., M.A. Marlindawati, and K. Shamsuddin, *Serological assessment of the establishment of herd immunity against measles in a health district in Malaysia.* BMC Infectious Diseases, 2016. **16(1) (no pagination)**.
19. Jablonka, A., et al., *Measles, mumps, rubella, and varicella seroprevalence in refugees in Germany in 2015.* Infection, 2016. **44**(6): p. 781-787.

20. Kader, C., et al., *Immunity of nursing students to measles, mumps, rubella, and varicella in Yozgat, Turkey.* American Journal of Infection Control, 2016. **44(1)**: p. e5-e7.
21. Tafuri, S., et al., *Monitoring the process of measles elimination by serosurveillance data: The Apulian 2012 study.* Vaccine, 2016. **34**(18): p. 2092-5.
22. Takemoto, K., et al., *Time-Series Analysis Comparing the Prevalence of Antibodies against Nine Viral Species Found in Umbilical Cord Blood in Japan.* Japanese Journal of Infectious Diseases, 2016. **69**(4): p. 314-8.
23. Toikkanen, S.E., et al., *Seroprevalence of Antibodies against Measles, Rubella and Varicella among Asylum Seekers Arriving in Lower Saxony, Germany, November 2014-October 2015.* International Journal of Environmental Research & Public Health [Electronic Resource], 2016. **13**(7): p. 30.
24. Gioula, G., et al., *Seroprevalence of measles in Northern Greece.* Acta Microbiologica Hellenica, 2017. **62(3)**: p. 145-150.
25. Jablonka, A., et al., *Measles, Rubella and Varicella IgG Seroprevalence in a Large Refugee Cohort in Germany in 2015: A Cross-Sectional Study.* Infectious Diseases & Therapy, 2017. **6**(4): p. 487-496.
26. Kader, C., et al., *Antibodies against vaccine preventable diseases in pregnant women measles, mumps, rubella, varicella and tetanus in Yozgat, Turkey.* Konuralp Tip Dergisi, 2017. **9(2)**: p. 29-34.
27. Koivisto, K., et al., *Immunity against vaccine-preventable diseases in Finnish pediatric healthcare workers in 2015.* Vaccine, 2017. **35**(12): p. 1608-1614.
28. Sutcliffe, C.G., et al., *Measles and Rubella Seroprevalence Among HIV-infected and Uninfected Zambian Youth.* Pediatric Infectious Disease Journal, 2017. **36**(3): p. 301-306.
29. Wang, X., et al., *Seroprevalence of Measles Antibodies and Predictors for Seropositivity among Chinese Children.* International Journal of Environmental Research & Public Health [Electronic Resource], 2017. **14**(6): p. 06.
30. Conway, J.J., et al., *Prevalence of inadequate immunity to measles, mumps, rubella, and varicella in MLB and NBA athletes.* Sports Health, 2018. **10**(5): p. 406-411.
31. Freidl, G.S., et al., *Immunity against measles, mumps, rubella, varicella, diphtheria, tetanus, polio, hepatitis A and hepatitis B among adult asylum seekers in the Netherlands, 2016.* Vaccine, 2018. **36**(12): p. 1664-1672.
32. Hachiya, M., et al., *Evaluation of nationwide supplementary immunization in Lao People's Democratic Republic: Population-based seroprevalence survey of anti-measles and anti-rubella IgG in children and adults, mathematical modelling and a stability testing of the vaccine.* PLoS ONE [Electronic Resource], 2018. **13**(3): p. e0194931.
33. Hubschen, J.M., et al., *IgG antibody prevalence suggests high immunization needs in newcomers to Luxembourg, 2012.* Vaccine, 2018. **36**(6): p. 899-905.
34. Izadi, S., S.M. Zahraei, and T. Mokhtari-Azad, *Seroprevalence of antibodies to measles and rubella eight months after a vaccination campaign in the southeast of Iran.* Human vaccines & Immunotherapeutics, 2018. **14**(6): p. 1412-1416.
35. Meng, Q.H., et al., *Seroprevalence of Maternal and Cord Antibodies Specific for Diphtheria, Tetanus, Pertussis, Measles, Mumps and Rubella in Shunyi, Beijing.* Scientific Reports, 2018. **8**(1): p. 13021.
36. Murray, A.F., et al., *Measles and Rubella Seroprevalence in Mother-Infant Pairs in Rural Nepal and the United States: Pre- and Post-Elimination Populations.* American Journal of Tropical Medicine & Hygiene, 2018. **99**(5): p. 1342-1345.
37. Scepanovic, P., et al., *Human genetic variants and age are the strongest predictors of humoral immune responses to common pathogens and vaccines.* Genome Medicine, 2018. **10(1) (no pagination)**.
38. Shoho, Y., et al., *Vaccination Status and Antibody Titers against Rubella and Measles among Japanese Female College Students Majoring in Childcare between 2015 and 2018.* Tohoku Journal of Experimental Medicine, 2018. **246**(2): p. 73-79.
39. Tomaskova, H., et al., *Serological survey of measles immunity in the Czech Republic, 2013.* Central European Journal of Public Health, 2018. **26**(1): p. 22-27.
40. Antona, D., et al., *Measles and rubella seroprevalence in a population of young adult blood donors, France 2013.* Epidemiology & Infection, 2019. **147**: p. e109.
41. Coppeta, L., et al., *Evaluation of immunity to measles in a cohort of Medical Students in Rome, Italy.* Vaccines, 2019. **7(4) (no pagination)**.

42. Gupta, M., et al., *Seroprevalence of measles, mumps & rubella antibodies among 5-10 years old children in north India.* Indian Journal of Medical Research, 2019. **149**(3): p. 396-403.
43. Hagstam, P., B. Bottiger, and N. Winqvist, *Measles and rubella seroimmunity in newly arrived adult immigrants in Sweden.* Infectious Diseases, 2019. **51**(2): p. 122-130.
44. Hayford, K., et al., *Measles and rubella serosurvey identifies rubella immunity gap in young adults of childbearing age in Zambia: The added value of nesting a serological survey within a post-campaign coverage evaluation survey.* Vaccine, 2019. **37**(17): p. 2387-2393.
45. Holka, J., K. Pawlak, and O. Ciepiela, *Seroprevalence of IgG antibodies against measles in a selected Polish population - Do we need to be re-vaccinated?* Central European Journal of Immunology, 2019. **44(4)**: p. 380-383.
46. Jung, J., et al., *Seroprevalence of Measles in Healthcare Workers in South Korea.* Infection & Chemotherapy, 2019. **51**(1): p. 58-61.
47. Khetsuriani, N., et al., *Measles and rubella seroprevalence among adults in Georgia in 2015: helping guide the elimination efforts.* Epidemiology & Infection, 2019. **147**: p. e319.
48. Marchi, S., et al., *Measles in pregnancy: a threat for Italian women?* Human Vaccines and Immunotherapeutics, 2019. **15(12)**: p. 2851-2853.
49. Odemis, I., et al., *Seroprevalence of measles, mumps, rubella, and varicella zoster virus antibodies among healthcare students: analysis of vaccine efficacy and cost-effectiveness.* Revista Espanola de Quimioterapia, 2019. **32**(6): p. 525-531.
50. Rasheed, M.A.U., et al., *Decreased humoral immunity to mumps in young adults immunized with MMR vaccine in childhood.* Proceedings of the National Academy of Sciences of the United States of America, 2019. **116**(38): p. 19071-19076.
51. Ristic, M., et al., *Sero-epidemiological study in prediction of the risk groups for measles outbreaks in Vojvodina, Serbia.* PLoS ONE [Electronic Resource], 2019. **14**(5): p. e0216219.
52. Santosh, K., et al., *Measles, mumps, and rubella: a cross-sectional study of susceptibility to vaccine-preventable diseases among young people in India. (Infectious diseases.).* Medical Journal Armed Forces India, 2019. **75**(1): p. 70-73.
53. Staehelin, C., et al., *Seroprotection rates of vaccine-preventable diseases among newly arrived Eritrean asylum seekers in Switzerland: a cross-sectional study.* Journal of Travel Medicine, 2019. **26**(6): p. 02.
54. Vos, R.A., et al., *Risk of measles and diphtheria introduction and transmission on Bonaire, Caribbean Netherlands, 2018.* American Journal of Tropical Medicine and Hygiene, 2019. **101(1)**: p. 237-241.
55. Vos, R.A., et al., *Seroepidemiology of Measles, Mumps and Rubella on Bonaire, St. Eustatius and Saba: The First Population-Based Serosurveillance Study in Caribbean Netherlands.* 2019. **7**(4): p. 01.
56. Wangchuk, S., et al., *Measles and rubella immunity in the population of Bhutan, 2017.* Vaccine, 2019. **37**(43): p. 6463-6469.
57. Wanlapakorn, N., et al., *Antibodies against measles and rubella virus among different age groups in Thailand: A population-based serological survey.* PLoS ONE [Electronic Resource], 2019. **14**(11): p. e0225606.
58. Adam, O., et al., *Seroprevalence of measles, mumps, and rubella and genetic characterization of mumps virus in Khartoum, Sudan.* International Journal of Infectious Diseases, 2020. **91**: p. 87-93.
59. Bechini, A., et al., *Immunization Status against Measles, Mumps, Rubella and Varicella in a Large Population of Internationally Adopted Children Referred to Meyer Children's University Hospital from 2009 to 2018.* 2020. **8**(1): p. 28.
60. Borocz, K., et al., *Application of a fast and cost-effective 'three-in-one' MMR ELISA as a tool for surveying anti-MMR humoral immunity: the Hungarian experience.* Epidemiology & Infection, 2020. **148**: p. e17.
61. Breakwell, L., et al., *Seroprevalence of chronic hepatitis B virus infection and immunity to measles, rubella, tetanus and diphtheria among schoolchildren aged 6-7 years old in the Solomon Islands, 2016.* Vaccine, 2020. **38**(30): p. 4679-4686.
62. Estofolete, C.F., et al., *Prevalence of measles antibodies in Sao Jose do Rio Preto, Sao Paulo, Brazil: a serological survey model.* Scientific Reports, 2020. **10**(3).

63. Feldstein, L.R., et al., *Vaccination coverage survey and seroprevalence among forcibly displaced Rohingya children, Cox's Bazar, Bangladesh, 2018: A cross-sectional study.* PLoS Medicine / Public Library of Science, 2020. **17**(3): p. e1003071.
64. Karadeniz, A. and E. Akduman Alasehir, *Seroepidemiology of hepatitis viruses, measles, mumps, rubella and varicella among healthcare workers and students: Should we screen before vaccination?* Journal of Infection and Public Health, 2020. **13**(4): p. 480-484.
65. Kwak, Y.G., et al., *Comparison of the Seroprevalence of Measles Antibodies among Healthcare Workers in Two Korean Hospitals in 2019.* Infection & Chemotherapy, 2020. **52**(1): p. 93-97.
66. Lucca, A., et al., *Lower Rate of Seropositivity to Measles among Young Healthcare Personnel in New York City.* Clinical Infectious Diseases, 2020. **71(12)**: p. 3241-3243.
67. Madi, N., et al., *Assessment of immune status against measles, mumps, and rubella in young Kuwaitis: MMR vaccine efficacy.* Journal of Medical Virology, 2020. **92**(8): p. 963-970.
68. Maltezou, H.C., et al., *Vaccination coverage and immunity levels against vaccine-preventable diseases in male Air Force recruits in Greece.* Vaccine, 2020. **38**(5): p. 1181-1185.
69. Minta, A.A., et al., *Seroprevalence of Measles, Rubella, Tetanus, and Diphtheria Antibodies among Children in Haiti, 2017.* American Journal of Tropical Medicine & Hygiene, 2020. **103**(4): p. 1717-1725.
70. Nakladalova, M., et al., *Measles immunity in a Czech tertiary care hospital.* Vaccine, 2020. **38(14)**: p. 2889-2892.
71. Ng, Y., et al., *Seroprevalence of vaccine-preventable diseases among children and adolescents in Singapore: Results from the National Paediatric Seroprevalence Survey 2018.* International Journal of Infectious Diseases, 2020. **92**: p. 234-240.
72. Nogareda, F., et al., *Measles and rubella IgG seroprevalence in persons 6 month-35 years of age, Mongolia, 2016.* Vaccine, 2020. **38**(26): p. 4200-4208.
73. Ogawa, T., et al., *Impact of vaccination on measles, mumps, and rubella antibody titers in Japanese healthcare workers: An observational study.* PLoS ONE, 2020. **15(3) (no pagination)**.
74. Zahraei, S.M., et al., *Seroprevalence of anti-rubella and anti-measles antibodies in women at the verge of marriage in Iran.* Vaccine, 2020. **38**(2): p. 235-241.
75. Zanella, B., et al., *Increasing Measles Seroprevalence in a Sample of Pediatric and Adolescent Population of Tuscany (Italy): A Vaccination Campaign Success.* 2020. **8**(3): p. 08.
76. Bianchi, F.P., et al., *Long-term immunogenicity after measles vaccine vs. wild infection: an Italian retrospective cohort study.* Human Vaccines and Immunotherapeutics, 2021. **17(7)**: p. 2078-2084.
77. Carcelen, A.C., et al., *Impact of a Measles and Rubella Vaccination Campaign on Seroprevalence in Southern Province, Zambia.* American Journal of Tropical Medicine & Hygiene, 2021. **104**(6): p. 2229-2232.
78. Coppeta, L., et al., *Seroprevalence for vaccine-preventable diseases among Italian healthcare workers.* Human vaccines & Immunotherapeutics, 2021. **17**(5): p. 1342-1346.
79. Frau, N., et al., *Seroepidemiology of Measles, Mumps, Rubella and Varicella in Italian Female School Workers: A Cross-Sectional Study.* 2021. **9**(10): p. 16.
80. Friedrich, N., et al., *Seroprevalence of Measles-, Mumps-, and Rubella-specific antibodies in the German adult population - cross-sectional analysis of the German Health Interview and Examination Survey for Adults (DEGS1).* The Lancet Regional Health. Europe, 2021. **7**: p. 100128.
81. Gupta, R., N. Saxena, and P. Gupta, *Determination of ELISA Reactive Mumps IgG Antibodies in MMR Vaccine Recipients in Comparison with MMR Vaccine Naive Children: a Cross Sectional Study.* Scripta Medica (Banja Luka), 2021. **52**(3): p. 174-180.
82. Khampanisong, P., et al., *Waning of maternal antibodies against measles suggests a large window of susceptibility in infants in lao people's Democratic republic.* Pathogens, 2021. **10(10) (no pagination)**.
83. Kim, C.J., et al., *Risk of Absence of Measles Antibody in Healthcare Personnel and Efficacy of Booster Vaccination.* 2021. **9**(5): p. 12.
84. Mikhail Kostinov, P., et al., *Gender differences in the level of antibodies to measles virus in adults.* Vaccines, 2021. **9(5) (no pagination)**.

85. Mahallawi, W.H. and N.A. Ibrahim, *Seroprevalence against the measles virus after vaccination or natural infection in an adult population in Madinah, Saudi Arabia.* Human vaccines & Immunotherapeutics, 2021. **17**(8): p. 2522-2529.
86. Muthiah, N., et al., *Dynamics of maternally transferred antibodies against measles, mumps, and rubella in infants in Sri Lanka.* International Journal of Infectious Diseases, 2021. **107**: p. 129-134.
87. Norman, F.F., et al., *Seroprevalence of vaccine-preventable and non-vaccine-preventable infections in migrants in Spain.* Journal of Travel Medicine, 2021. **28**(4): p. 01.
88. von Linstow, M.L., et al., *Immunity to vaccine-preventable diseases among paediatric healthcare workers in Denmark, 2019.* Euro Surveillance: Bulletin Europeen sur les Maladies Transmissibles = European Communicable Disease Bulletin, 2021. **26**(17).
89. Xaydalasouk, K., et al., *Age-stratified seroprevalence of vaccine-preventable infectious disease in Saravan, Southern Lao People's Democratic Republic.* International Journal of Infectious Diseases, 2021. **107**: p. 25-30.
90. Yang, X., et al., *Evaluation of measles vaccination coverage in Lincang City, Yunnan Province, China.* Human Vaccines and Immunotherapeutics, 2021. **17(9)**: p. 3145-3152.
91. Yoo, Y., et al., *Seroprevalence of measles, mumps, rubella, and varicella-zoster antibodies in new female nurses in the Republic of Korea.* Annals of Occupational & Environmental Medicine, 2021. **33**: p. e19.
92. Carcelen, A.C., et al., *Leveraging a national biorepository in Zambia to assess measles and rubella immunity gaps across age and space.* Scientific Reports, 2022. **12**(1): p. 10217.
93. Hase, R., Y. Niiyama, and H. Mito, *Evaluation of the seroprevalence of measles, rubella, mumps, and varicella and the requirement for additional vaccination based on the JSIPC guidelines among emergency medical technicians at eight fire stations in Narita, Japan: a project review.* Human vaccines & Immunotherapeutics, 2022. **18**(1): p. 1989922.
94. Ichimura, Y., et al., *Effectiveness of immunization activities on measles and rubella immunity among individuals in East Sepik, Papua New Guinea: A cross-sectional study.* IJID Regions (Online), 2022. **3**: p. 84-88.
95. Lin, M.Y., H.H. Shao, and M.T. Tsou, *Measles immunity in medical center staff after changes in national and local hospital vaccination policies.* BMC Infectious Diseases, 2022. **22**(1): p. 427.
96. Murhekar, M.V., et al., *Evaluating the effect of measles and rubella mass vaccination campaigns on seroprevalence in India: a before-and-after cross-sectional household serosurvey in four districts, 2018-2020.* The Lancet Global Health, 2022. **10**(11): p. e1655-e1664.
97. Nokhodian, Z., et al., *A Nationwide Study on the Seroprevalence of Measles, Mumps, and Rubella in Iranian Children and Adolescents.* Iranian Journal of Public Health, 2022. **51**(2): p. 409-415.
98. Quach, H.Q., et al., *Seroprevalence of Measles Antibodies in a Highly MMR-Vaccinated Population.* Vaccines, 2022. **10(11) (no pagination)**.
99. Virachith, S., et al., *Susceptibility to Vaccine-Preventable Diseases in Four Districts of Xaysomboun Province, Lao People's Democratic Republic.* 2022. **10**(3): p. 17.
100. Chung, H., et al., *Causes of a Low Measles Seroprevalence among Young Healthcare Workers in Korea.* Infection & Chemotherapy, 2023. **55**(3): p. 388-393.
101. Gusmao, C., et al., *Seroprevalence and prevention of hepatitis B, measles and rubella among healthcare workers in Dili, Timor-Leste.* The Lancet Regional Health. Southeast Asia, 2023. **13**: p. 100133.
102. Kia, M., et al., *Investigation of the Seroprevalence of Antimeasles Immunoglobulin G Antibody in Students at Shiraz University of Medical Sciences.* Viral Immunology, 2023. **36**(6): p. 424-428.
103. Miyano, S., et al., *Comparison of population-based measles-rubella immunoglobulin G antibody prevalence between 2014 and 2019 in Lao People's Democratic Republic: Impacts of the national immunization program.* International Journal of Infectious Diseases, 2023. **129**: p. 70-77.
104. Pedranti, M., et al., *Measles and Rubella Seroprevalence Among Children and Adolescents of Cordoba, Argentina: A Cross-Section Study in the Context of the Elimination Program.* Viral Immunology, 2023. **36**(6): p. 429-434.

105. Santacruz-Sanmartin, E., Hincapié-Palacio, D., Ochoa-Acosta, J. E., Buitrago-Giraldo, S., and Ospina, M. C., *Measles Serostatus in Mothers and Their Newborns in Antioquia, Colombia: Implications for Measles Elimination.* American Journal of Tropical Medicine and Hygiene, 2023. **108(1)**: p. 93-100.
106. Gadallah, M., et al., *Seroprevalence of rubella antibodies among adult Egyptian females aged 20-30 years. Is there a need for rubella vaccination?* Central European Journal of Public Health, 2014. **22**(4): p. 282-6.
107. Lo Giudice, D., et al., *Congenital rubella syndrome and immunity status of immigrant women living in southern Italy: a cross-sectional, seroepidemiological investigation.* Travel Medicine & Infectious Disease, 2014. **12**(3): p. 253-7.
108. Chua, Y.X., et al., *An epidemiological assessment towards elimination of rubella and congenital rubella syndrome in Singapore.* Vaccine, 2015. **33**(27): p. 3150-7.
109. Gallone, M.S., et al., *Lack of immunity against rubella among Italian young adults.* BMC Infectious Diseases, 2017. **17**(1): p. 199.
110. Nobrega, Y.K.M., et al., *Rubella Seropositivity in Pregnant Women After Vaccination Campaign in Brazil's Federal District.* Viral Immunology, 2017. **30**(9): p. 675-677.
111. Edirisuriya, C., et al., *Australian rubella serosurvey 2012-2013: On track for elimination?* Vaccine, 2018. **36**(20): p. 2794-2798.
112. Siira, L., et al., *Response to third rubella vaccine dose.* Human vaccines & Immunotherapeutics, 2018. **14**(10): p. 2472-2477.
113. Crooke, S.N., et al., *Seroprevalence and durability of rubella virus antibodies in a highly immunized population.* Vaccine, 2019. **37**(29): p. 3876-3882.
114. Viswanathan, R., et al., *Comparison of two commercial ELISA kits for detection of rubella specific IgM in suspected congenital rubella syndrome cases and rubella IgG antibodies in a serosurvey of pregnant women.* Diagnostic Microbiology & Infectious Disease, 2019. **94**(3): p. 243-247.
115. Coppeta, L., et al., *Rubella immunity among Italian female healthcare workers: a serological study.* International Journal of Environmental Research and Public Health, 2020. **17**(21).
116. Motaze, N.V., et al., *Rubella seroprevalence using residual samples from the South African measles surveillance program: a cross-sectional analytic study.* Human vaccines & Immunotherapeutics, 2020. **16**(11): p. 2656-2662.
117. Patic, A., et al., *Seroepidemiological study of rubella in Vojvodina, Serbia: 24 years after the introduction of the MMR vaccine in the national immunization programme.* PLoS ONE [Electronic Resource], 2020. **15**(1): p. e0227413.
118. Shahapur, P.R. and V. Kandi, *Seroprevalence of Rubella Virus-specific Antibodies in Women and the Diagnostic Efficacy of Enzyme-linked Immunoassay and Rapid Immunochromatographic Tests.* Cureus, 2020. **12**(3): p. e7246.
119. Shashank, S., et al., *Rubella seroprevalence among Indian female medical and nursing students at a tertiary care teaching institute and its correlation with socioeconomic status.* Indian Journal of Community Medicine, 2020. **45**(2): p. 246-247.
120. Gorun, F., et al., *Prevalence of Rubella Antibodies among Fertile Women in the West of Romania, 18 Years after the Implementation of Immunization.* 2021. **9**(2): p. 29.
121. Sasaki, H., et al., *Persistence of Anti-Rubella Immunoglobulin G Antibody Titers in Young Adults Involved in a Short-Term Periodic Immunization in Japan.* Japanese Journal of Infectious Diseases, 2021. **74**(5): p. 473-476.
122. Shanmugasundaram, D., et al., *Burden of congenital rubella syndrome (CRS) in India based on data from cross-sectional serosurveys, 2017 and 2019-20.* PLoS Negl Trop Dis, 2021. **15**(7): p. e0009608.
123. Toizumi, M., et al., *Rubella seroprevalence among mothers and incidence of congenital rubella three years after rubella vaccine introduction in Vietnam.* Human vaccines & Immunotherapeutics, 2021. **17**(9): p. 3156-3161.
124. Trevisan, A., et al., *Rubella Serosurvey Among Future Healthcare Workers.* Frontiers in Public Health, 2021. **9**: p. 741178.
125. Armah, N.B., et al., *Rubella virus IgM and IgG antibodies with avidity in pregnant women and outcomes at a tertiary facility in Ghana.* PLoS ONE, 2022. **17**(12).
126. Bassal, R., et al., *The Concordance between Mumps and Rubella Sero-Positivity among the Israeli Population in 2015.* Vaccines, 2022. **10(7) (no pagination)**.

127. Ibrahim, N.A. and W.H. Mahallawi, *Rubella Humoral Immunity Among the Saudi Population of Madinah in the Western Region of Saudi Arabia.* Viral Immunology, 2022. **35**(5): p. 375-380.
128. Lavrentieva, I.N., et al., *A Herd Immunity to Rubella Virus in Selected Geographical Regions.* Russian Journal of Infection and Immunity, 2022. **12(5)**: p. 902-908.
129. Hashemi, S.M.A., et al., *The frequency of IgG anti-varicella and anti-rubella antibodies in female students of Shiraz University of Medical Sciences, Iran.* Infezioni in Medicina, 2023. **31(4)**: p. 533-538.
130. Wang, X., et al., *Do adolescents need a rubella vaccination campaign? Rubella serosurvey among healthy children in Hangzhou, China.* Human vaccines & Immunotherapeutics, 2023. **19**(2): p. 2254536.
